# Spectroscopic, X‐Ray Crystallographic, and Hirshfeld Surface Analyses for the Investigation of Intermolecular Interactions in Carboxamide Hydrazone Hybrids

**DOI:** 10.1002/open.202500276

**Published:** 2025-08-03

**Authors:** Nabila A. Kheder, Mostafa E. Salem, Saied M. Soliman, Ismail A. Elhaty, Naglaa S. Mahmoud, Mohamed Abdel‐Megid, Kamal M. Dawood

**Affiliations:** ^1^ Department of Chemistry Faculty of Science Cairo University Giza 12613 Egypt; ^2^ College of Science Imam Mohammad Ibn Saud Islamic University (IMSIU) Riyadh 11623 Saudi Arabia; ^3^ Department of Chemistry Faculty of Science Alexandria University Ibrahimia Alexandria 21321 Egypt; ^4^ Department of Nutrition and Dietetics Faculty of Health Sciences Istanbul Gelisim University 34310 Istanbul Turkey; ^5^ Department of Industrial Pharmacy College of Pharmaceutical Sciences and Drug Manufacturing Misr University for Science and Technology Giza P.O. 12566 Egypt

**Keywords:** antibacterial activity, antifungal activity, Hirshfeld calculations, hydrazone‐carboxamide hybrid, intermolecular interactions, X‐ray crystal structure

## Abstract

The current study reports a convenient synthetic approach to carboxamide hydrazone hybrids **2a, b**. Their chemical structures are investigated using infrared, nuclear magnetic resonance, and mass spectroscopies. In addition, the 3D structure of **2**a is explored using single‐crystal X‐ray analysis, while the important intermolecular contacts are described based on the Hirshfeld analysis. Fukui function, highest occupied molecular orbital‐lowest unoccupied molecular orbital, molecular electrostatic potential, and Mulliken charge analysis reveal distinct electrophilic and nucleophilic regions of hydrazone **2a**, and support its stability and moderate chemical reactivity. Their antimicrobial potency is also evaluated against six microbial strains. Compound **2b** shows significant antibacterial activity against *Staphylococcus aureus* compared to Gentamicin.

## Graphical abstract

Two novel carboxamide hydrazone hybrids are synthesized and characterized. Structural, spectroscopic, and computational analyses reveal dominant hydrogen bonding, π–π stacking, and reactive sites. Compound 2b exhibits potent antibacterial activity against *Staphylococcus aureus*, highlighting its potential as a cost‐effective therapeutic agent. Insights aid future drug design efforts.

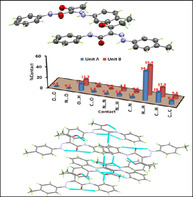

## 1 Introduction

Hydrogen bonds are important in chemical and biological systems because they stabilize structures and facilitate reactions.^[^ ^1^, ^2^, ^3^ ^]^ The NH…O intramolecular hydrogen bond interactions are crucial in bioactive molecules and medications.^[^ ^4^ ^]^ Hydrogen bonding (HB) interactions are among many noncovalent interactions’ most directional and dynamic. These interactions are essential in our lives; for example, HB is accountable for folding proteins, self‐complementarity of nucleic acids, and the low density of frozen water. The HB interactions are due to electrostatics and polarization forces. Electrostatic forces in HB are directional based on the electrostatic potential of the N–H and O atoms.^[^ ^5^, ^6^, ^7^ ^]^ They can be enhanced by increasing partial charges on the donor and acceptor atoms, and the strength of these forces reduces to the minimum with increasing H…O distances. Polarization refers to the ability of the HB acceptor to rearrange electron density to better participate in HB. By measuring the bond lengths and bond angles in HB systems, it can be concluded that shorter HB distances and HB angles approaching 170°–180° lead to stronger HB interactions. Therefore, the interaction forces require high linearity and decrease significantly with deviation from optimal HB geometry and increasing distance.^[^ ^5^, ^6^, ^7^ ^]^

**Figure 1:**
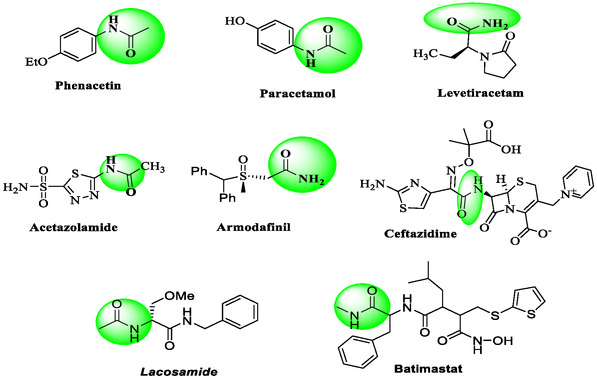
Examples of marketed drugs containing carboxamide function.

Spectroscopic techniques, such as infrared (IR) and ^1^H NMR spectroscopies, can also be employed in the characterization.^[^ ^8^ ^]^ The presence of the carboxamide group has a tangible effect on enhancing the biological potencies of organic molecules that are employed in clinically approved drugs, where the carboxamide group includes the hydrogen‐bond donor (NH) and the hydrogen‐bond acceptor (C = O) functions.^[^ ^9^, ^10^, ^11^, ^12^ ^]^ The carboxamide function is the backbone structural unit of some commercial drugs as represented in **Figure** **1**, such as phenacetin (pain‐relieving drug), paracetamol (treatment of fever and mild to moderate pain), levetiracetam (treatment of epilepsy), acetazolamide (treatment of glaucoma and epilepsy), armodafinil (treatment of excessive daytime sleepiness), ceftazidime (treatment of some bacterial infections) batimastat (antimetastatic drug), and lacosamide (treatment of partial‐onset seizures). In addition, compounds containing carboxamide function demonstrated a wide range of pharmacological activities, such as anthelmintic,^[^ ^13^ ^]^ antitubercular,^[^ ^14^ ^]^ and antitrypanosomal potencies.^[^ ^15^ ^]^

Given the acknowledged importance of the carboxamide scaffold, we continued our investigation into synthesizing hydrazone‐carboxamide hybrids as bioactive compounds.^[^ ^16^, ^17^, ^18^, ^19^, ^20^ ^]^ The structures and HB interactions of the synthesized hybrids were investigated using different spectroscopic tools. In addition, the 3D structure of one of the newly synthesized compounds is explored using single‐crystal X‐ray analysis, while the important intermolecular contacts are described based on the Hirshfeld analysis. In addition, the antimicrobial potency of compounds **2a** and **2b** was evaluated.

## 2 Results and Discussion

### 2.1 Chemistry and Characterization

**Scheme 1:**
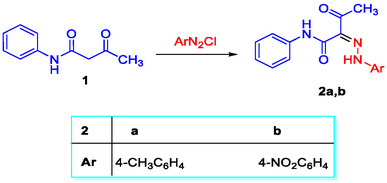
Structure of the carboxamide derivatives **2a** and **2b**.

The coupling of 3‐oxo‐*N*‐phenylbutanamide **1** with the diazonium salts of *p*‐toluidine and *p*‐nitroaniline in methanol containing sodium acetate and sodium hydroxide mixture afforded the corresponding hydrazone derivatives **2a** and **2b** in 35 and 63% yields, respectively.^[^ ^21^ ^]^ In anticipation of the low yields of the previous method, the same reactions were repeated in ethanol buffered with sodium acetate, where compounds **2a** and **2b** were isolated in high yields, 85% and 82%, respectively (**Scheme** **1**).

**Figure 2:**
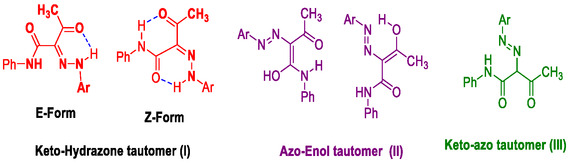
Tautomeric forms of compounds **2**.

As shown in **Figure** **2**, compounds **2a** and **2b** can be formulated in three possible tautomeric structures, **I–III**. Because tautomers have different acceptor‐donor characteristics, they can interact with molecules differently, leading to variations in their biological activities.^[^ ^22^, ^23^, ^24^ ^]^ So, the chemical structures of the synthesized compounds and the hydrogen bond interactions were investigated using X‐ray and spectral analysis.

**Figure 3:**
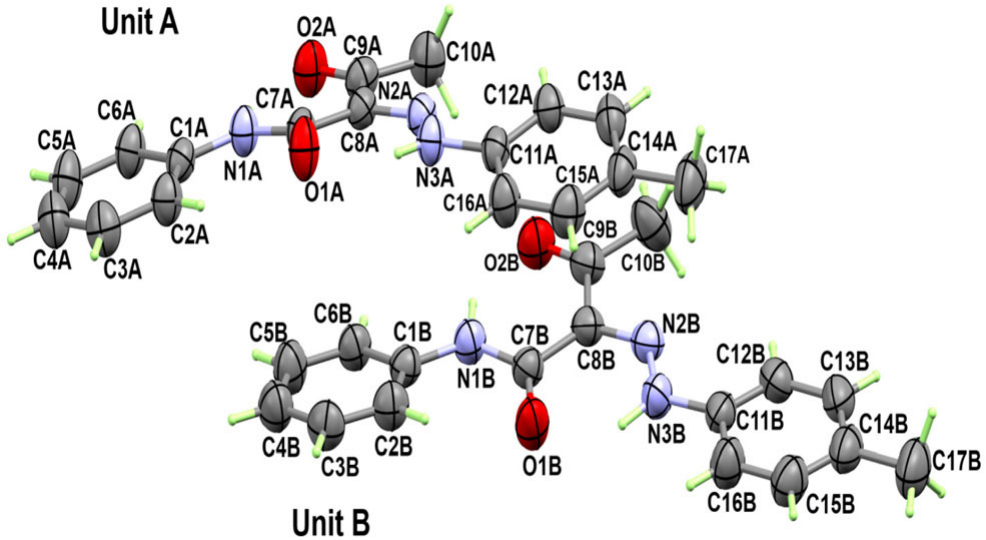
Molecular structure of **2a** with anisotropic displacement ellipsoids drawn at 50% probability level.

For example, in IR spectroscopy, the vibrational frequencies of the hydrogen bond donor and acceptor functional groups can change when a hydrogen bond is present.^[^ ^25^, ^26^, ^27^ ^]^ In the IR spectral data of **2a** and **2b**, two carbonyls and two NH groups’ stretching bands moved to lower wave numbers. These changes confirmed their presence in the *Z* structure of the keto‐hydrazone form, which is stabilized by two intramolecular hydrogen bonds. In addition, X‐ray analysis of **2a** (**Figure** **3**) provides another evidence for this result.

Also, the changes in proton chemical shifts in ^1^H NMR spectroscopy can be used to indicate HB interactions.^[^ ^28^, ^29^ ^]^ This bond lowers the electron density around the hydrogen atom, raising its chemical shift value.

For example, ^1^H NMR spectra of **2a** and **2b** showed only two singlet signals at *δ* 11.25–11.30 and 14.06–15.85 ppm; these results indicate the presence of only one tautomeric form, which may be (**I**) or (**II**) form. These signals were shifted to a lower field due to hydrogen bond interactions.^[^ ^30^, ^31^, ^32^, ^33^, ^34^, ^35^, ^36^ ^]^

Moreover, ^13^C NMR spectra of **2a** and **2b** revealed two carbon signals at *δ* 161.06–161.99 and 198.38–199.97, which are close to the reported values of hydrogen‐bonded amidic^[^ ^36^ ^]^ and ketonic^[^ ^37^, ^38^ ^]^ carbons. The presence of two signals of carbonyl carbons indicated the presence of only one tautomeric form, and low chemical shifts of these carbons could be attributed to conjugation and hydrogen bond interactions.^[^ ^39^ ^]^

The spectral results and single‐crystal X‐ray analysis confirmed that these compounds adopt the *Z* structure of the keto‐hydrazone form.

### 2.2 X‐Ray Structure Description

The molecular structure of **2a** is shown in Figure 3, while the detailed crystal data are listed in **Table** **1**. Compound **2a** crystallized in triclinic space group *P‐1* with two chemically equal but crystallographically independent molecules, A and B, per asymmetric unit. For molecule A, the twist angle is 13.38(3)°, while for molecule B, the corresponding value is 9.37(3)°.

**Table 1:** Crystal data and structure refinement for hydrazone **2a**.

CCDC	2,391,225
Empirical formula	C_17_H_17_N_3_O_2_
Formula weight	295.34
Temperature/K	296(2)
Crystal system	Triclinic
Space group	*P‐1*
a/Å	8.0586(5)
b/Å	12.0229(8)
c/Å	16.9468(9)
*α*/°	71.801(4)
*β*/°	83.300(4)
*γ*/°	84.316(4)
Volume/Å^3^	1545.74(17)
Z	4
*ρ*_calc_g/cm^3^	1.269
μ/mm^−1^	0.690
F(000)	624.0
Crystal size/mm^3^	0.2 × 0.18 × 0.11
Radiation	CuK*α* (*λ* = 1.54178)
2*Θ* range for data collection/°	5.512 to 133.322
Index ranges	−9 ≤ *h* ≤ 9, −14 ≤ *k* ≤ 14, −20 ≤ *l* ≤ 19
Reflections collected	35,411
Independent reflections	5461 [*R*_int_ = 0.0681, *R*_sigma _= 0.0438]
Data/restraints/parameters	5461/0/417
Goodness‐of‐fit on F^2^	1.019
Final R indexes [I >= 2*σ* (I)]	R_1 _= 0.0523, wR_2 _= 0.1312
Final R indexes [all data]	R_1 _= 0.0830, wR_2 _= 0.1570
Largest diff. peak/hole / e Å^−3^	0.21/−0.19

The selected bond lengths and angles are listed in **Table** **2**.

**Table 2:** Selected bond lengths [Å] and angles [°] for compound **2a**.

Bond lengths			
O1A‐C7A	1.233(3)	O1B‐C7B	1.232(3)
N2A‐N3A	1.298(3)	N2B‐N3B	1.308(3)
C1A‐C6A	1.387(3)	C1B‐C6B	1.379(3)
C5A‐C6A	1.380(4)	C5B‐C6B	1.381(3)
**Bond angles**			
C1A‐N1A‐C7A	128.7(2)	C1B‐N1B‐C7B	128.3(2)
N1A‐C1A‐C2A	124.2(2)	N1B‐C1B‐C2B	123.8(2)
C1A‐C2A‐C3A	119.7(2)	C1B‐C2B‐C3B	119.6(2)
C4A‐C5A‐C6A	120.9(2)	C4B‐C5B‐C6B	120.8(2)

**Figure 4:**
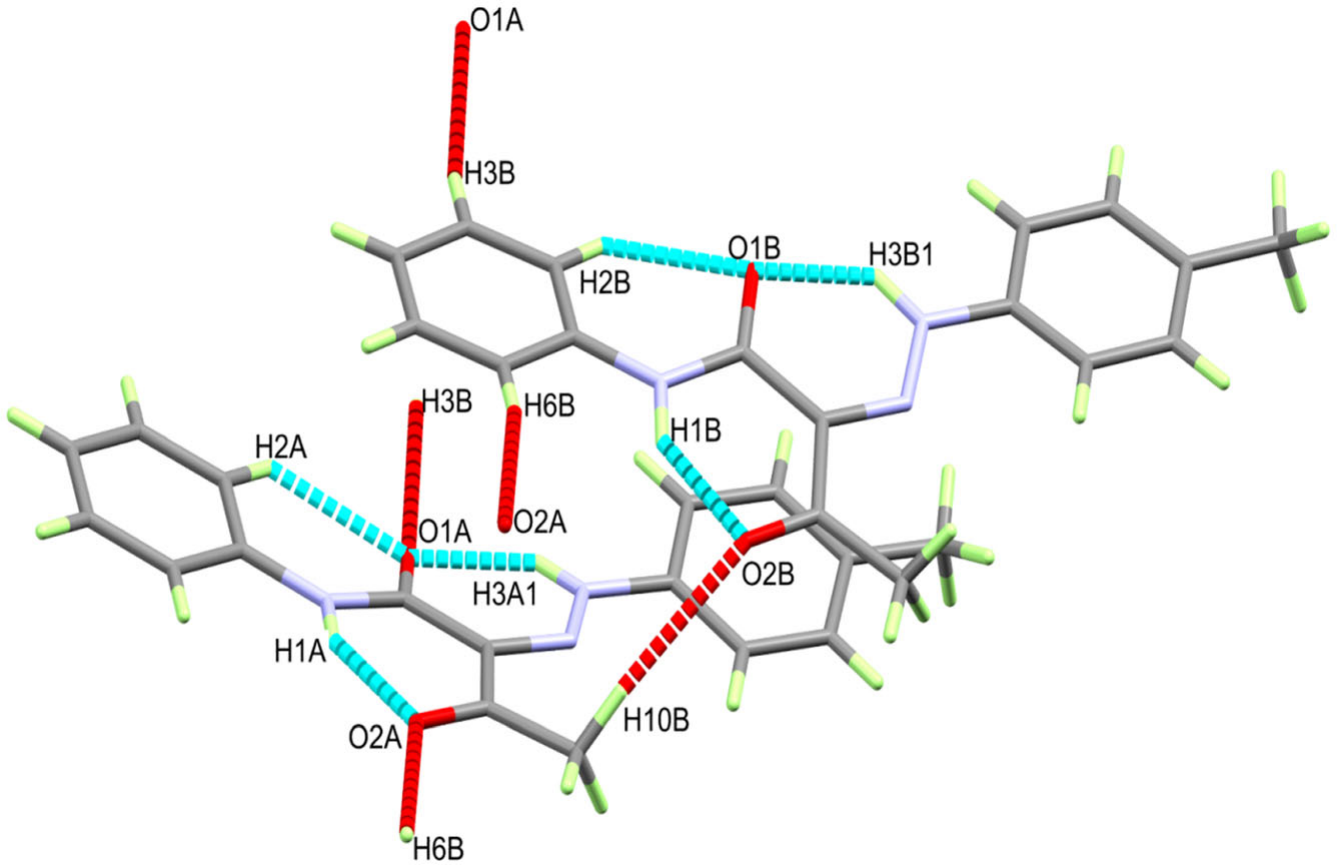
Intramolecular (turquoise) and intermolecular (red) interactions for compound **2a**.

It is clear from the X‐ray structure analysis of the hydrazone **2a** that both molecules in the asymmetric formula exist in the sterically hindered *Z‐*form, which is stabilized by the intramolecular NH…O HB interactions presented in **Figure** **4**. There are three intramolecular noncovalent interactions for each molecular unit. In unit **A**, the N1A‐H1A…O2A, N3A‐H3A1…O1A and C2A‐H2A…O1A intramolecular interactions have donor to acceptor distances of 2.661(2), 2.580(2), and 2.888(3) Å, respectively. In unit B, the corresponding values for the N1B‐H1B…O2B, N3B‐H3B1…O1B, and C2B‐H2B…O1B interactions are 2.669(3), 2.558(2), and 2.879(3) Å, respectively.

A list of these interactions and their geometric parameters is depicted in **Table** **3**.

**Table 3:** Hydrogen bonds for compound **2a** [Å and °].

D‐H…A	d(D‐H)	d(H…A)	d(D…A)	<(DHA)	Symm. code
N3B‐H3B1…O1B	0.896(7)	1.858(7)	2.558(2)	133.3(6)	
N1A‐H1A…O2A	0.893(8)	1.907(6)	2.661(2)	141.0(3)	
N1B‐H1B…O2B	0.896(8)	1.915(7)	2.669(3)	140.7(3)	
N3A‐H3A1…O1A	0.879(8)	1.900(8)	2.580(2)	132.9(8)	
C2A‐H2A…O1A	0.93	2.29	2.888(3)	122	
C2B‐H2B…O1B	0.93	2.32	2.879(3)	118	
C3B‐H3B…O1A	0.93	2.59	3.495(3)	165	‐x, 2‐y, 1‐z
C6B‐H6B…O2A	0.93	2.56	3.471(3)	165	1‐x, 1‐y, 1‐z
C10A‐H10B…O2B	0.96	2.60	3.544(3)	168	

**Figure 5:**
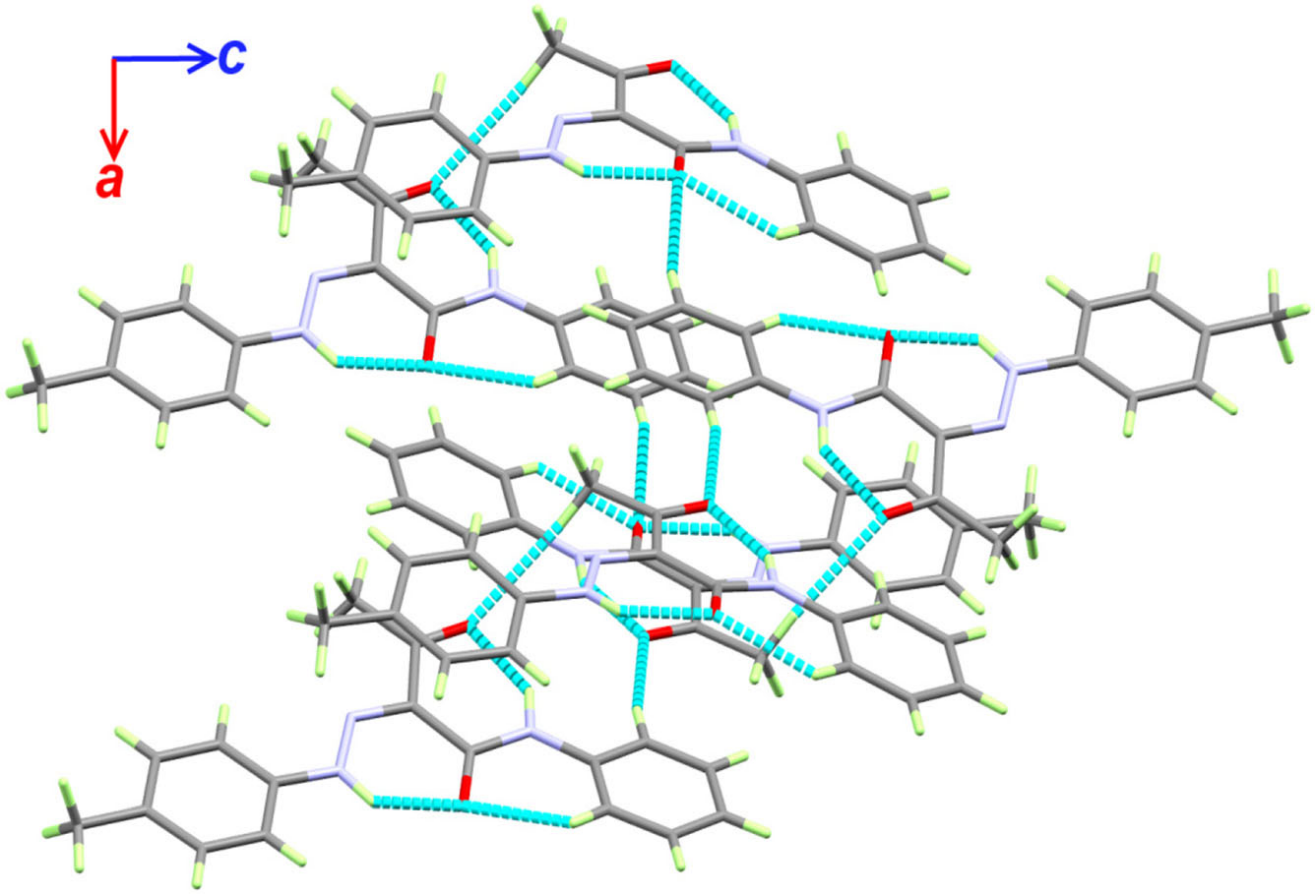
Packing scheme along *ac*‐plane for compound **2a**.

Intermolecular interactions connect the molecules in the crystal belonging to the C3B‐H3B…O1A, C6B‐H6B…O2A, and C10A‐H10B…O2B interactions which have C3B…O1A, C6B…O2A, and C10A…O2B distances of 3.495(3), 3.471(3), and 3.544(3) Å, respectively. An illustration of the packing scheme is shown in **Figure** **5**.

**Figure 6:**
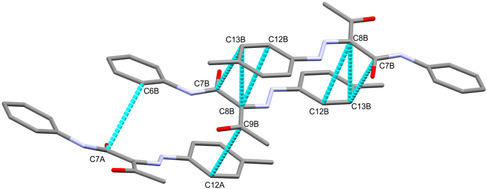
The *π–* *π* stacking interactions in **2a**.

In addition, there are short C…C interactions that belong to *π*–*π* stacking interactions. A list of these contacts and their interaction distances is given in **Table** **4**, while the presentation of these *π*–*π* stacking interactions is shown in **Figure** **6**.

**Table 4:** Hydrogen bonds for hydrazone‐carboxamide hybrid **2a** [Å and °].

Contact	Distance	Symm. code
C7B‐C13B	3.493	‐x, 1‐y, 2‐z
C8B‐C12B	3.395	‐x, 1‐y, 2‐z
C8B‐C13B	3.469	‐x, 1‐y, 2‐z
C6B‐C7A	3.495	
C9B‐C12A	3.493	

### 2.3 Hirshfeld Surface Analysis

**Figure 7:**
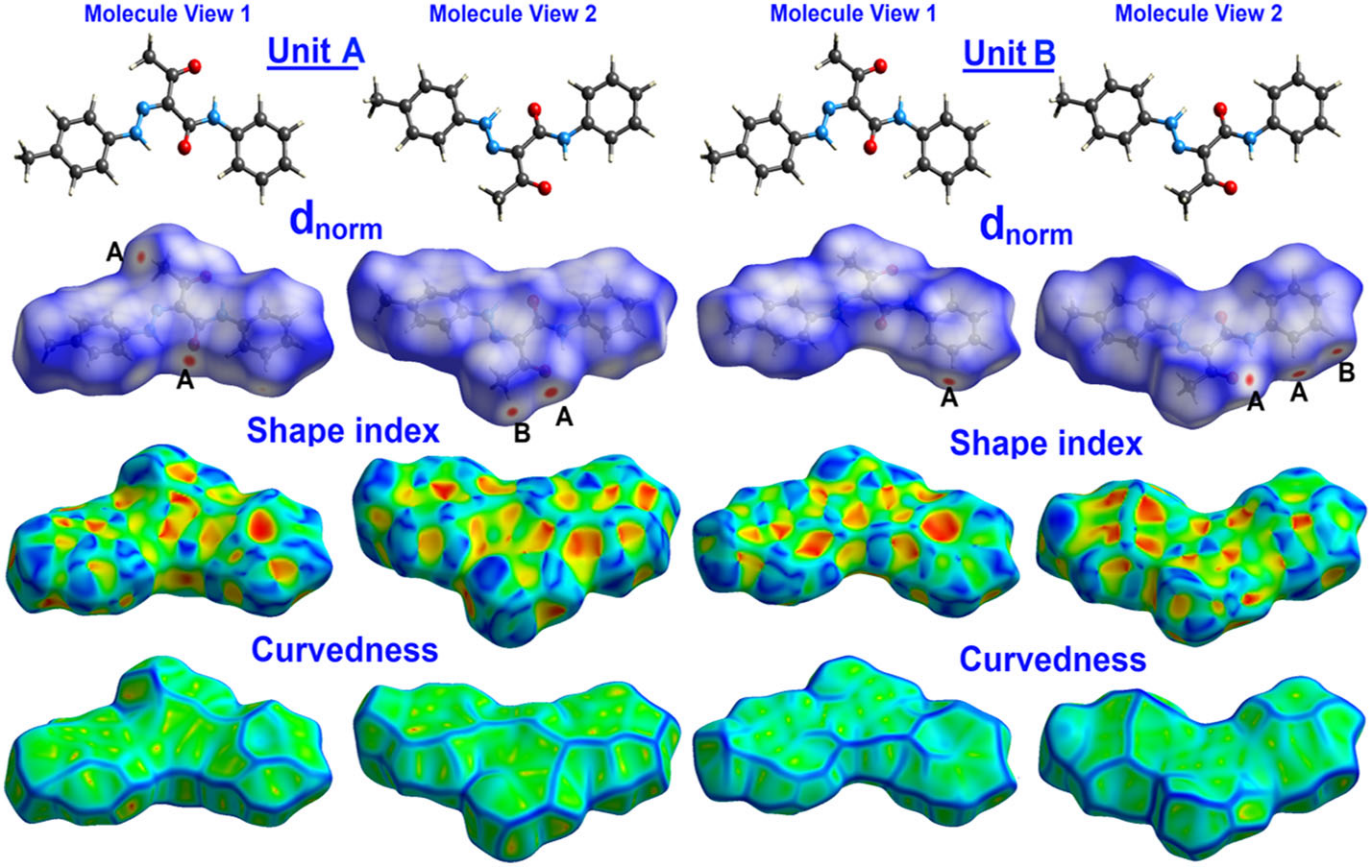
Hirshfeld maps for hydrazone **2a**; A) O…H and B) H…H.

Further analysis for molecular packing was performed using Hirshfeld calculations. The *d*_norm_, shape index, and curvedness maps are presented in **Figure** **7**. It is clear that the two units in the asymmetric formula are shared in two important intermolecular interactions, which are the O…H and H…H contacts.

**Figure 8:**
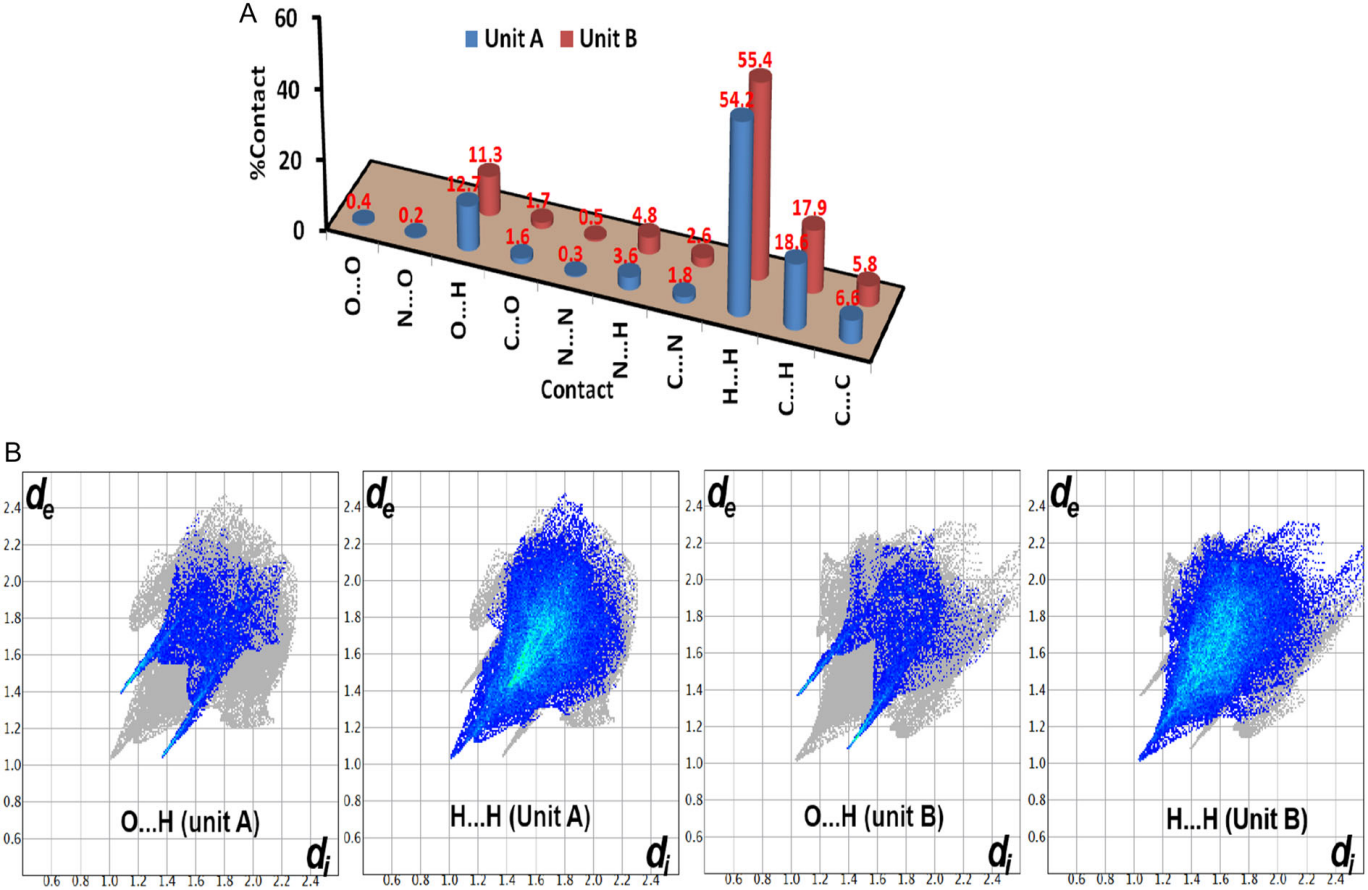
Noncovalent interactions **A**) and decomposed fingerprint plots for the short contacts **B**) in compound **2a**.

All intermolecular interactions that contributed to the molecular packing of **2a** are presented in **Figure** **8A**. In unit **A**, the most important contacts are the O…H and H…H interactions, which also occurred in unit **B** but with different percentages. The %O…H contacts are 12.7 and 11.3%, respectively (Figure 8A). The O1B…H3A (2.547 Å), O1A…H3B (2.441 Å), O2A…H6B (2.416 Å), O2B…H6A (2.573 Å), and O2B…H10B (2.478 Å) are the shortest contacts. In both units, the H…H interactions contribute the most to the molecular packing. The %H…H interactions are 54.2 and 55.4% for units **A** and **B**, respectively. Also, the H…H contacts appeared as red spots indicating H…H distances shorter than twice the vdWs radii sum of hydrogen atoms. The shortest H…H distance is H10A…H5B (2.051 Å). The presence of these interactions has an important role in the stability of the crystal structure.

Also, the decomposed fingerprint plots indicated the presence of C…C contacts (5.8–6.6%), revealing the presence of *π–* *π* stacking interactions. This fact is further confirmed by the presence of red and blue triangles in the shape index map and the flat green area in curvedness (Figure 8). It is worth noting that the O…H and H…H interactions appeared as characteristic sharp spikes revealing another good evidence on their importance in the molecular packing of hydrazone‐carboxamide hybrid **2a** (Figure 8).

### 2.4 Fukui Functions

**Figure 9:**
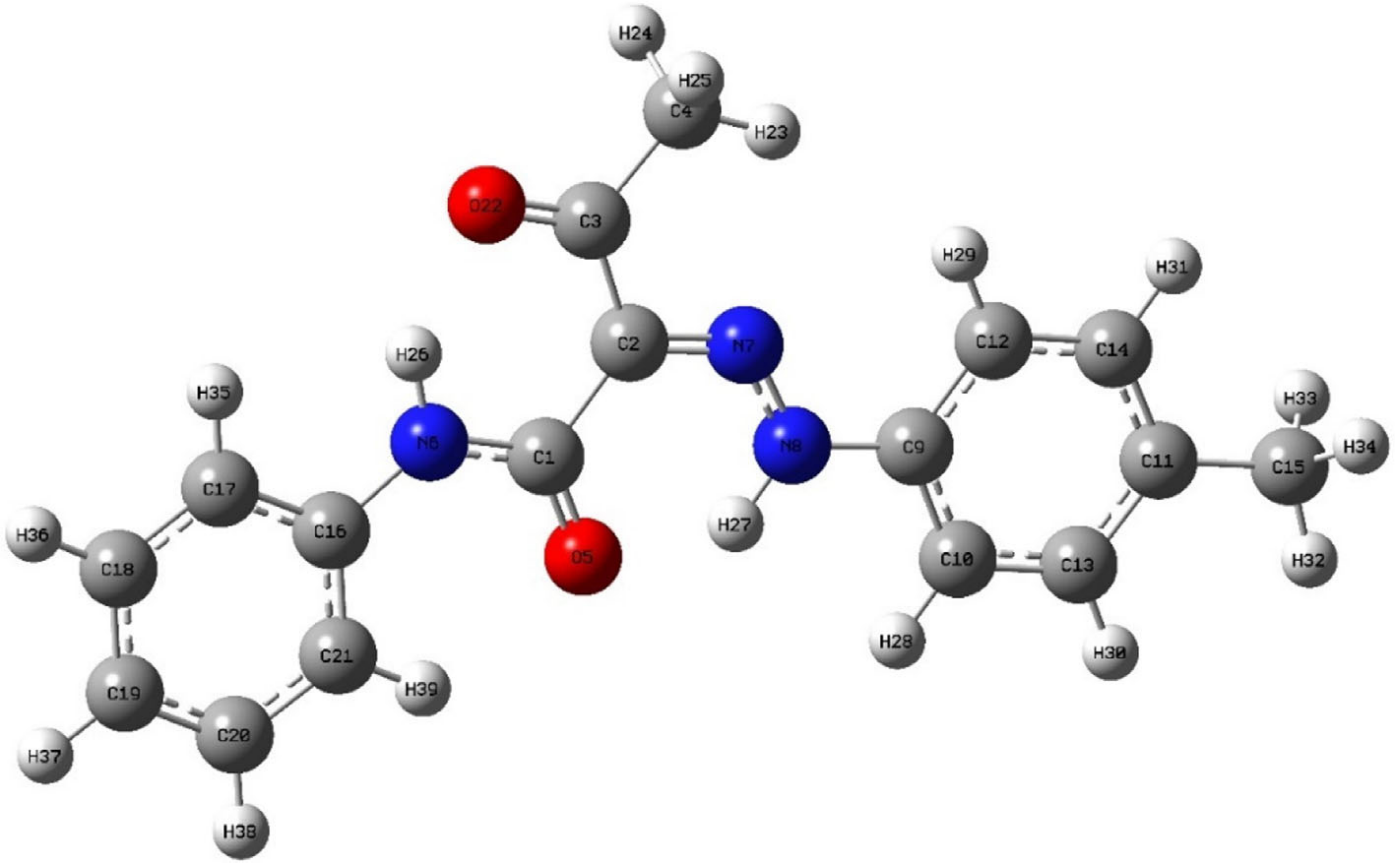
The optimized structure of hydrazone **2a**.

The chemical reactivity and site selectivity of the optimized hydrazone **2a** (**Figure** **9**) were studied using Fukui function through conceptual density functional theory (CDFT).^[^ ^40^ ^]^ Fukui function is a significant computational tool used to investigate the most favorable sites in the molecule for electrophilic ($f_{k}^{-})$, nucleophilic ($f_{k}^{+})$, and radical ($f_{k}^{0})$ attack using the following equations^[^ ^41^ ^]^

(1) $$f_{k}^{-}=q_{k}\left(N_{0}\right)-q_{k}\left(N_{0}-1\right)$$

(2) $$f_{k}^{+}=q_{k}\left(N_{0}+1\right)-q_{k}\left(N_{0}\right)$$

(3) $$f_{k}^{0}=\frac{1}{2}(f_{k}^{+}+f_{k}^{-})$$

where *q*_k_ represents the electronic population of atom k, while (N_0_), (N_0_ + 1), and (N_0_ ‐ 1) represent the molecule in its neutral, anionic, and cationic states, respectively.

Additionally, the dual descriptor index (Δf(r)) showed better prediction of the reactivity of atomic sites and their selectivity towards nucleophilic or electrophilic attack.^[^ ^42^ ^]^ It is the difference between $f_{k}^{+}$ and $f_{k}^{-}$, and is calculated using the following equation

(4) $$\text{Δ}f\left(r\right)=[f^{+}\left(r\right)-f^{-}\left(r\right)]$$

The site shows preference to nucleophilic attack if Δf(r)>0, while it shows preference to electrophilic attack Δf(r)<0.

The calculated values of $f_{k}^{-}$, $f_{k}^{+}$, $f_{k}^{0}$, and Δf(r) are shown in **Table** **5**. It has been found that N7, O22, C3, O5, and C12 have high values of $f_{k}^{+}$ and positive values of Δf(r) indicating the most possible sites for the nucleophilic attack. On the other hand, C19, C11, C2, N6, and N8 have high $f_{k}^{-}$ values with negative Δf(r) values predicting the most favorable sites for electrophilic attack.

**Table 5:** Fukui analysis of hydrazone **2a**.

Atom	***f***_**k**_^**−**^	***f***_**k**_^**+**^	***f***_**k**_^**0**^	Δ*f*(r)
C1	−0.0138	0.0193	0.0028	0.0055
C2	−0.0111	0.0722	0.0306	0.0611
C3	0.0068	0.0585	0.0327	0.0517
C4	−0.0052	−0.0075	0.0064	0.0023
O5	0.0453	0.049	0.0471	0.0037
N6	0.0979	0.0241	0.061	−0.0738
N7	0.036	0.1615	0.0987	0.1255
N8	0.1096	0.0584	0.084	−0.0513
C9	0	−0.0143	0.0072	0.0143
C10	0.0604	0.0297	0.045	−0.0307
C11	0.0956	0.0815	0.0886	−0.0142
C12	0.0565	0.0485	0.0525	−0.008
C13	0.0029	0.0193	0.0111	0.0164
C14	0.0052	0.0073	0.0063	0.0021
C15	−0.0191	−0.0132	0.0162	−0.0059
C16	0.0029	−0.0191	0.0081	0.0162
C17	0.0408	0.016	0.0284	−0.0248
C18	0.0087	0.0123	0.0105	0.0035
C19	0.0905	0.045	0.0677	−0.0455
C20	−0.0056	0.0095	0.002	0.004
C21	0.0663	0.017	0.0417	−0.0493
O22	0.0443	0.0981	0.0712	0.0538
H23	−0.0045	−0.0002	0.0023	−0.0044
H24	0.0195	0.0285	0.024	0.009
H25	0.0198	0.0285	0.0242	0.0087
H26	0.0235	−0.0005	0.0115	−0.023
H27	−0.0012	0.016	0.0074	0.0148
H28	0.0124	0.0133	0.0129	0.0009
H29	0.0165	0.0017	0.0091	−0.0148
H30	0.0249	0.0211	0.023	−0.0038
H31	0.0249	0.0202	0.0226	−0.0047
H32	0.0144	0.0137	0.014	−0.0007
H33	0.0267	0.0205	0.0236	−0.0062
H34	0.0265	0.0206	0.0236	−0.0059
H35	0.0179	0.0034	0.0106	−0.0145
H36	0.025	0.0148	0.0199	−0.0102
H37	0.0217	0.0156	0.0186	−0.0061
H38	0.0228	0.0134	0.0181	−0.0093
H39	−0.0056	−0.0039	0.0048	−0.0016

### 2.5 Frontier Molecular Orbital (FMO) Analysis

FMO was employed to study the electronic behavior, chemical reactivity, and overall stability of the target molecule using CDFT calculations. Highest occupied molecular orbital (HOMO) and lowest unoccupied molecular orbital (LUMO) energies reflect the molecule's ability to donate electrons or accept electrons, respectively. The difference between HOMO and LUMO energies (energy gap) may indicate the molecule's reactivity and kinetic stability. For hydrazone **2a**, HOMO and LUMO are lying at −6.035 and −2.477 eV, respectively, while the energy gap (Δ*E*) was obtained at 3.559 eV.

**Figure 10:**
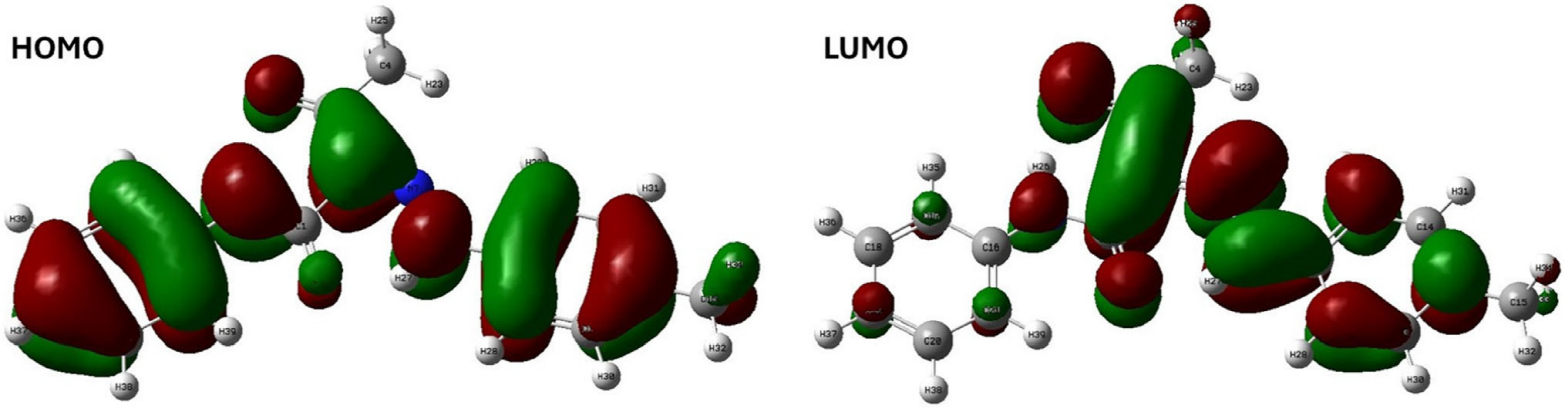
The HOMO and LUMO levels for hydrazone **2a**.

The spatial distribution of the HOMO is primarily localized over electron‐rich regions and *π*‐donating systems. The spatial distribution of the HOMO orbital is predominantly localized over most of the molecular framework as shown in **Figure** **10**, whereas LUMO is mainly localized on electron‐deficient regions and *π*‐acceptor systems.

In addition, to further study the reactivity of the title molecule, various global chemical reactivity descriptors were calculated from the HOMO and LUMO energies using CDFT calculations. The relative electrophilicity and relative nucleophilicity descriptors are employed as alternative local reactivity indices to predict the most reactive electrophilic and nucleophilic sites in the molecule. The ionization potential (IP) was approximated as the negative of the HOMO energy, while the electron affinity (Ea) was approximated as the negative values of the LUMO energy. The HOMO–LUMO energy gap is directly related to global chemical hardness (*η*) and can be used as an indicator of molecular stability and reactivity. Other global reactivity descriptors including hardness, softness, electronegativity, and chemical potential were calculated using the following equations:^[^ ^43^ ^]^

Global chemical hardness,

(5) $$\eta =\frac{\left(IP-Ea\right)}{2}$$

Chemical potential,

(6) $$\mu =-\frac{\left(IP+Ea\right)}{2}$$

Global chemical softness,

(7) $$S=\frac{1}{\eta }$$

Electronegativity,

(8) $$\chi =\frac{\left(IP+Ea\right)}{2}$$

Based on the above‐mentioned equations, the IP, electron affinity (Ea), chemical potential (μ), chemical hardness (*η*), softness (S), and electronegativity (*χ*) of hydrazone **2a** were found 6.035, 2.477, −3.568, 1.779 eV, 0.562 eV^−1^, and 4.256 eV, respectively. The relatively wide HOMO–LUMO gap and the descriptors values indicate that the molecule has a moderate electronic hardness and electron‐accepting ability, supporting its classification as a kinetically stable and less reactive molecule.

### 2.6 Molecular Electrostatic Potential (MEP) Analysis

**Figure 11:**
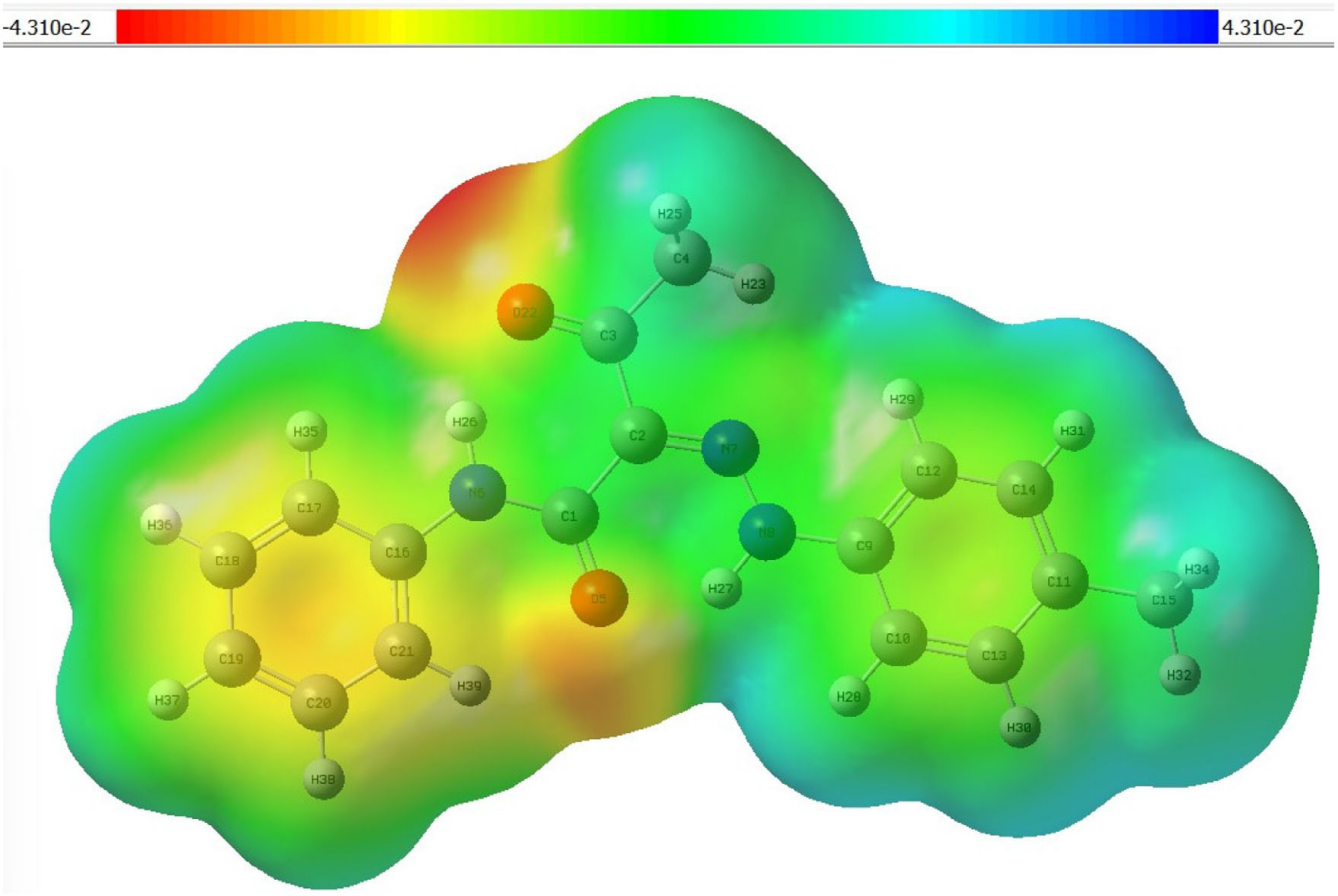
The MEP map for hydrazone **2a**.

The MEP map of the optimized hydrazone **2a** was computed using the B3LYP/6‐311++G(d, p) method to visualize the spatial distribution of electrostatic potential across the molecule, as shown in **Figure** **11**. The resulted MEP surface indicates the chemically reactive regions within the molecule, including sites prone to electrophilic and nucleophilic interactions, as well as potential intra‐ and intermolecular forces, in particular HB based on the electronic density and charge distribution.^[^ ^44^ ^]^ The color gradient from red to blue in the MEP map indicates the difference in the electrostatic potential. In hydrazone **2a**, the most negative regions are observed around oxygen atoms (**O5** and **O22**) of the carbonyl groups, represented in red on the MEP surface with a maximum negative potential value of −4.310e −2 at **O22** indicating it as the most preferred site for electrophilic attack. Aromatic rings appear in yellow to yellowish green representing regions of relatively moderate negative potential. On the other hand, areas with electropositive appear in light blue are primarily located around the hydrogen atoms on the aliphatic side chains, with a peak positive potential of + 4.310e−2, suggesting possible sites for nucleophilic attack.

### 2.7 Mulliken Charge Analysis

The Mulliken atomic charge analysis is used to obtain atomic charge distribution across a molecule which provides a significant insight into the electronic structure, intramolecular interactions, and potential reactive sites within this molecule. Since atomic charges affect the molecule's physicochemical properties such as dipole moment, polarizability, and charge transfer behavior, so Mulliken analysis helps understanding the donor–acceptor dynamics in the molecule.

**Figure 12:**
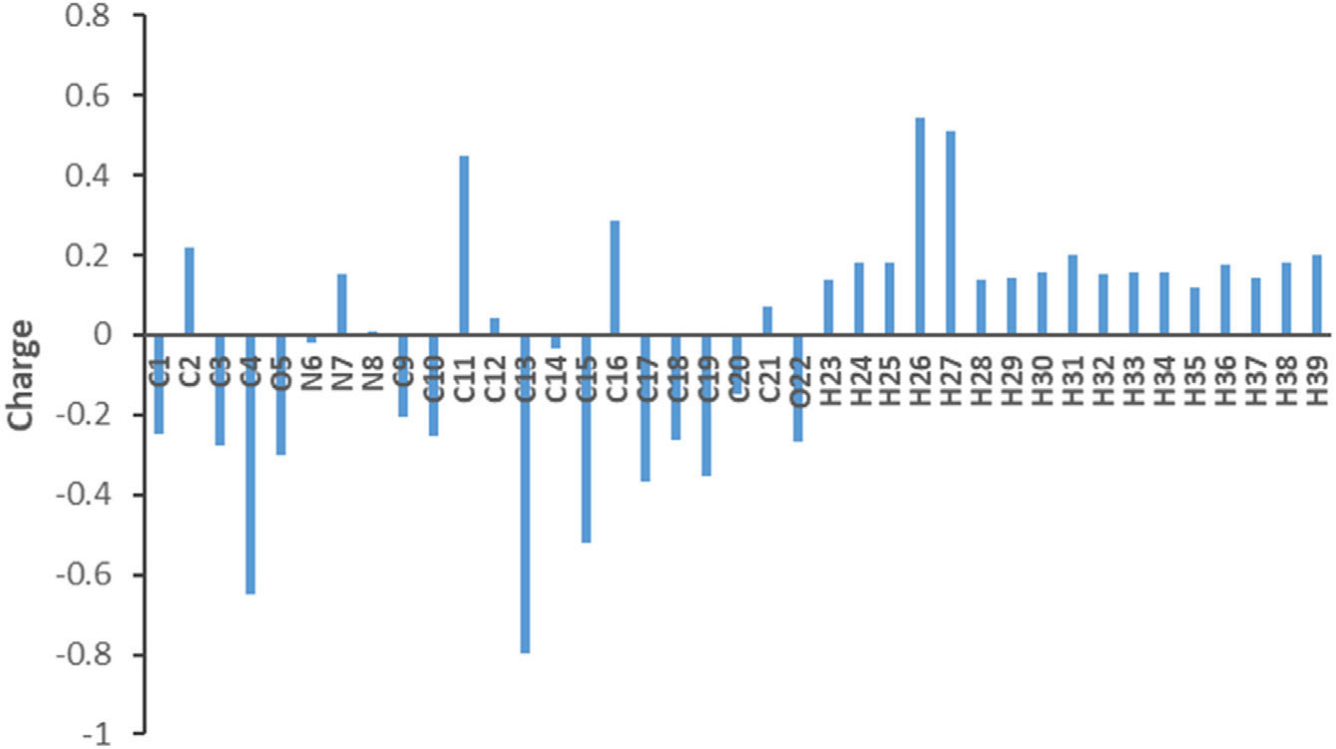
Plot of Mulliken charge for hydrazone **2a** molecule.

Mulliken atomic charges of hydrazone **2a** are presented in **Table** **6** and visualized in **Figure** **12**. The results show that oxygen atoms **O5** (−0.299) and **O22** (−0.268), as well as nitrogen **N6** (−0.020) showed high negative charges, therefore, they are likely to participate in HB or act as targets for electrophilic attack. Additionally, the carbon atom **C3** (−0.278) also exhibits significant negative charges, likely due to delocalization across the aromatic system. On the other hand, hydrogen atoms, specifically **H26** (+0.545) and **H27** (+0.510) bonded to electronegative atom (nitrogen), carry significant positive charges, making them probable donors in HB interactions. These findings support the predictions of the molecule's reactivity, especially in terms of nucleophilic and electrophilic interactions.

**Table 6:** Mulliken charges for hydrazone **2a** molecule.

Atom	Charge	Atom	Charge
C1	−0.248533	C21	0.071347
C2	0.219809	O22	−0.268009
C3	−0.2778	H23	0.136049
C4	−0.650026	H24	0.179281
O5	−0.298902	H25	0.179126
N6	−0.019633	H26	0.544722
N7	0.151382	H27	0.510342
N8	0.008447	H28	0.140014
C9	−0.205394	H29	0.143872
C10	−0.255658	H30	0.156538
C11	0.44713	H31	0.198821
C12	0.043252	H32	0.153487
C13	−0.799854	H33	0.15735
C14	−0.033365	H34	0.157865
C15	−0.51828	H35	0.120476
C16	0.288436	H36	0.17731
C17	−0.370034	H37	0.14377
C18	−0.264094	H38	0.182051
C19	−0.354488	H39	0.200587
C20	−0.147392		

### 2.8 The In Vitro Antibacterial and Antifungal Assessments

**Figure 13:**
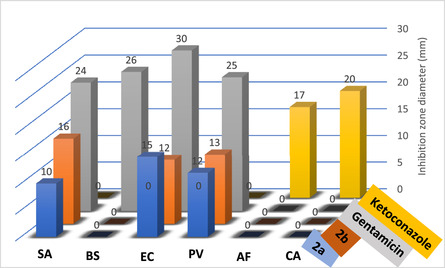
Antimicrobial activity results of **2a** and **2b**.

The carboxamide function is the backbone structural unit of some antimicrobial medications, including penicillins, tetracyclines*,* ceftazidime, and isoniazid. Also, the hydrazone product obtained by coupling the diazonium salt of sulfapyridine with *N*‐(2‐methylphenyl)‐3‐oxobutanamide inhibits HIV integrase.^[^ ^45^ ^]^ Therefore, the **2a** and **2b**'s antibacterial activities were tested using the well diffusion method^[^ ^46^ ^]^ against *Staphylococcus aureus* ATCC 25,923 (**SA**), *Bacillus subtilis* RCMB 015 (1) NRRL B‐543 (**BS**), *Escherichia coli* ATCC 25,922 (**EC**), and *Proteus vulgaris* RCMB 004 (1) ATTC 13,315 (**PV**) using Gentamicin as a reference drug. Their antifungal activity was also tested against *Aspergillus fumigatus* (RCMB 0022008) (**AF**) and *Candida albicans* RCMB 005003 (1) ATCC 10,231 (**CA**), and their ability was compared to antifungal Ketoconazole. The results are reported as inhibition zone diameter (IZD) (mm), as outlined in **Figure** **13**.

The antibacterial assays showed that carboxamide derivatives **2a** and **2b** displayed significant inhibitory efficiency towards *Staphylococcus aureus*, *Escherichia coli*, and *Proteus vulgaris* strains. It was also revealed that compound **2b** was highly potent against *Staphylococcus aureus,* which can be attributed to the nitro group as its electron‐withdrawing effect enhances the compound binding to enzymes, as previously reported.^[^ ^47^ ^]^

## 3 Conclusion

A convenient method for the synthesis of two carboxamide hydrazone hybrids **2a, b** is described. Their chemical structures and HB interactions were investigated using different spectroscopic tools. The crystal structure of **2a** is dominated by the large amount of H…H interactions (54.2–55.4%). In addition, there is a significant amount of O…H contacts, which contributed by 11.3–12.7%. Moreover, the characteristic features from shape index and curvedness about the *π*‐*π* stacking interactions are observed where the %C…C contacts are 6–6.9%. Computational analyses for **2a**, including DFT‐based HOMO–LUMO mapping and Fukui function evaluation, revealed localized reactive centers and confirmed the molecule's moderate reactivity and electronic stability. Compounds **2a** and **2b** were evaluated for their antimicrobial activity, and the results showed considerable antibacterial activity, especially compound **2b**, which demonstrated high activity against *Staphylococcus aureus*. This study contributes valuable insights into relations between HB interactions within molecules and their spectral data. Also, it could help advance the design of new economic therapeutic agents for treating *Staphylococcus aureus* infections.

## 4 Experimental Section

4.1

4.1.1

##### Chemistry: General Remarks

A Gallenkamp melting point apparatus was used to determine the melting points. The infrared spectra were recorded on KBr disks on a Pye Unicam SP 3300 and Shimadzu FT‐IR 8101 PC infrared spectrophotometers. The nuclear magnetic resonance (NMR) spectra were recorded on Bruker‐500 (running at 500 MHz for ^1^H and 125 MHz for ^13^C) (King Khalid University, KSA) or on Varian Mercury Vx‐300 BB (running at 300 MHz for ^1^H). Chemical shifts were related to that of the solvents. Elemental analyses were carried out at the Microanalytical Center of Cairo University, Egypt. Antimicrobial assays were carried out in the Regional Center for Mycology and Biotechnology, Al‐Azhar University, Cairo, Egypt. 3‐oxo‐*N*‐phenylbutanamide (**1**)^[^ ^48^ ^]^ was synthesized as reported in the literature.

##### Chemistry: Synthetic Procedure of Hydrazone‐Carboxamide Hybrids **2a** and **2b**

To a stirred mixture of butanamide **1** (1.77g, 10 mmol) and CH_3_CO_2_Na·3H_2_O (1.3 g, 10 mmol) in EtOH (50 mL), was added the appropriate arene diazonium salts [prepared by diazotizing *p*‐toluidine *or p*‐nitroaniline (10 mmol) in HCl (6 M, 6 mL) with NaNO_2_ solution (0.7 g, 10 mmol) in H_2_O (3 mL)]. The addition process was conducted portion‐wise with shaking for 30 min at 0–5°C. After addition, the reaction mixture was left to shake for 4 h. The obtained solid matter was filtered off and then recrystallized from EtOH to produce the corresponding hydrazones **2a** and **2b**, respectively.

##### Chemistry: (Z)‐3‐Oxo‐N‐Phenyl‐2‐(2‐(p‐Tolyl)hydrazinylidene)butanamide (**2a**)

Crystals suitable for X‐ray analysis were obtained by slow evaporation of EtOH at room temperature.

Yield (2.51g, 85%), mp. 132°C (EtOH), (Lit. Mp. 124–126°C^21^, IR (KBr): v 3228–3134 (NH, very weak and broad), 1654 (ketone C = O), 1593 (amide C = O) cm^−1^; ^1^H NMR (300 MHz, DMSO‐*d*_*6*_) *δ*: 2.29 (s, 3H, CH_3_), 2.45 (s, 3H, CH_3_), 7.14 (m, 1H), 7.21 (d, 2H, *J* = 7.8 Hz), 7.37 (m, 4H), 7.63 (d, 2H, *J* = 7.8 Hz), 11.25 (s, 1H, NH), 14.06 (s, 1H, NH); ^13^C NMR (300 MHz, DMSO‐*d*_*6*_) *δ* = 20.52, 25.78, 115.85, 120.22, 124.41, 126.13, 129.01, 130.02, 134.48, 137.27, 139.45, 161.99, 198.38; MS m/z (%), 295 (M^+^, 4.13%). Anal. Calcd for C_17_H_17_N_3_O_2_ (295.34): C, 69.14; H, 5.80; N, 14.23. Found: C, 69.22; H, 5.69; N, 14.12%.

##### Chemistry: (Z)‐2‐(2‐(4‐Nitrophenyl)hydrazinylidene)‐3‐Oxo‐N‐Phenylbutanamide (**2b**)

Yield (2.67g, 82%), mp 214°C (EtOH), (Lit. Mp. 213–216^21^); IR (KBr) v 3228–3165 (NH, very weak and broad), 1664 (ketone C = O), 1594 (amide C = O) cm^−1^; ^1^H NMR (500 MHz, DMSO‐*d*_*6*_) *δ*: 2.66 (s, 3H, CH_3_), 7.17–7.75 (m, 7H), 8.09 (d, 1H, *J* = 10.0 Hz), 8.29 (d, 1H, *J* = 10.0 Hz), 11.30 (s, 1H, NH), 15.85 (s, 1H, NH); ^13^C NMR (DMSO‐*d*_*6*_) *δ* 26.56, 116.97, 120.78, 123.66, 125.02, 126.16, 129.06, 130.02, 135.73, 135.79, 137.02, 138.26, 161.06, 199.97. MS m/z (%), 327 (M^+^ + 1, 19.57%). Anal. Calcd for C_16_H_14_N_4_O_4_ (326.31): C, 58.89; H, 4.32; N, 17.17. Found: C, 58.81; H, 4.32; N, 17.14.

##### X‐Ray Structure Determination

Intensity data were collected with a Bruker X8 prospector diffractometer using Cu‐K*α* radiation at room temperature. The structure was solved by direct methods and was expanded using Fourier techniques. The nonhydrogen atoms were refined anisotropically. SHELXTL and SHELXL‐2017/1 were used for structure solution and refinement.^[^ ^49^ ^]^ The H atoms for the NH groups were located from the residual electron‐density map. All carbon‐bound hydrogen atoms were positioned geometrically, with C–H distances of methyl and aromatic groups being 0.96 and 0.93 Å respectively, and the isotropic displacement parameters of hydrogen atoms were refined with *U*_iso_(H) = 1.2*U*_eq_(C) except for hydrogen atoms from methyl groups, where *U*_iso_(H) = 1.5*U*_eq_(C) was used. **CCDC 2,391,225** contains the *supplementary* crystallographic data for this compound, which can be obtained free of charge from the Cambridge Crystallographic Data Centre via www.ccdc.cam.ac.uk/data_request/cif. Crystal data of **2a** are depicted in Table 1.

##### Hirshfeld Surface Analysis

The topology analyses were performed using the Crystal Explorer 17.5 program.^[^ ^50^ ^]^

##### Fukui Functions

Fukui function was calculated to determine the nucleophilic and electrophilic sites of hydrazone derivative **2a**. All calculations were conducted using the Gaussian 09W and visualized on GaussView 6.0. Geometry optimization of this compound was carried out under neutral conditions using CDFT with B3LYP/6‐311++G(d, p) basis set.^[^ ^51^ ^]^ Fukui function values were analyzed using the UCA‐FUKUI V 2.0 tool.^[^ ^52^ ^]^ In addition, NBO program integrated with Gaussian 09W was used for natural bond orbital (NBO) analysis using POP = NBO keyword.^[^ ^53^ ^]^ The NBO calculations were used to investigate the electron density distribution, donor–acceptor interactions, and atomic charges, providing complementary insight into the electronic structure and reactive behavior of the molecule. These results support the identification of reactive sites predicted by Fukui function analysis. All computations were performed on a laptop equipped with a 13th Gen Intel Core i7‐1360P processor (2.20 GHz) and 16 GB RAM.

##### FMO Analysis

FMOs analysis is widely used to explain the electronic properties of organic compounds such as their chemical reactivity and stability. The HOMO, LUMO, and the band gap (*Δ*E) energies of hydrazone **2a** were calculated using the aforementioned CDFT method and Gaussian 09W software. The *Δ*E value indicates the compound's chemical stability and reactivity. A molecule with a high frontier energy gap has low chemical reactivity and high kinetic stability.

##### MEP Surface

The MEP surface was used to predict reactive sites for electrophilic (red and yellow) and nucleophilic (blue) attack for hydrazone **2a** through the three‐dimensional charge distribution of this molecule. This analysis was carried out using the aforementioned DFT method and Gaussian 09W software. In addition, these electrostatic features provide insight into reactive sites and potential intermolecular interactions, such as HB involving electronegative atoms like oxygen and nitrogen.^[^ ^44^ ^]^

##### Mulliken Atomic Charge Analysis

For deeper insight into the internal charge distribution in hydrazone derivative **2a**, Mulliken atomic charge analysis was carried out using the previously specified DFT method and basis set. The obtained results provide valuable information about electron localization, which influences properties such as molecular polarity, dipole moment, electronic configuration, and both intra‐ and intermolecular interactions.

Additionally, the chemical behavior and stability of hydrazone **2a** were evaluated by calculating global reactivity descriptors, including electronegativity (*χ*), chemical potential (μ), global hardness (*η*), global softness (S), and the electrophilicity index (*ω*). These parameters were derived from frontier molecular orbital energies (HOMO and LUMO) in line with CDFT.

##### The In Vitro Antimicrobial Assessment

Compounds **2a** and **2b** were tested in vitro for their antimicrobial activity against six infectious agents*.* Gentamicin was used as a standard drug for Gram‐positive and Gram‐negative bacteria. Ketoconazole was used as a standard drug for fungi strains. DMSO was used as a negative control. The prepared compounds were tested at a 10 mg/mL concentration against both bacterial and fungal strains.

##### The In Vitro Antimicrobial Assessment: Method of Testing

Screening tests regarding the inhibition zone were conducted using the well diffusion method^[^ ^42^ ^]^ [more details in SI file].

## Conflict of Interest

The authors declare no conflict of interest.

## Supporting information

Supplementary Material

## Data Availability

The data that support the findings of this study are available in the supplementary material of this article.
