# Supplementary material for: Spectroscopic, X‐Ray Crystallographic, and Hirshfeld Surface Analyses for the Investigation of Intermolecular Interactions in Carboxamide Hydrazone Hybrids
Source: ChemistryOpen. 2025 Aug 3;14(12):e202500276. doi: 10.1002/open.202500276 (PMC12680582; doi:10.1002/open.202500276)

Spectroscopic, X-ray Crystallographic, and Hirshfeld Surface Analyses for the Investigation of Intermolecular Interactions in Carboxamide Hydrazone Hybrids

Nabila A. Kheder[a], Mostafa E. Salem*[b], Saied M. Soliman[c], Ismail A. Elhaty* [d], Naglaa S. Mahmoud[e], Mohamed Abdel-Megid[b], and Kamal M. Dawood[a]

Antimicrobial evaluation

Method of testing:

Screening tests regarding the inhibition zone were conducted using the well diffusion method[42] The inoculum suspension was prepared from colonies grown overnight on an agar plate and inoculated into Mueller-Hinton broth (fungi using malt broth). A sterile swab was immersed in the suspension to inoculate Mueller-Hinton agar plates (fungi using malt agar plates and bacteria using nutrient agar plates). The compounds were dissolved in dimethyl sulfoxide (DMSO) with different concentrations (10, 5, 2.5, …… mg/ml) to determine MIC value. The inhibition zone was measured around each well after 48h at 28 ^{o}C for fungi and 24h at 37 ^{o}C for bacteria

Spectral data

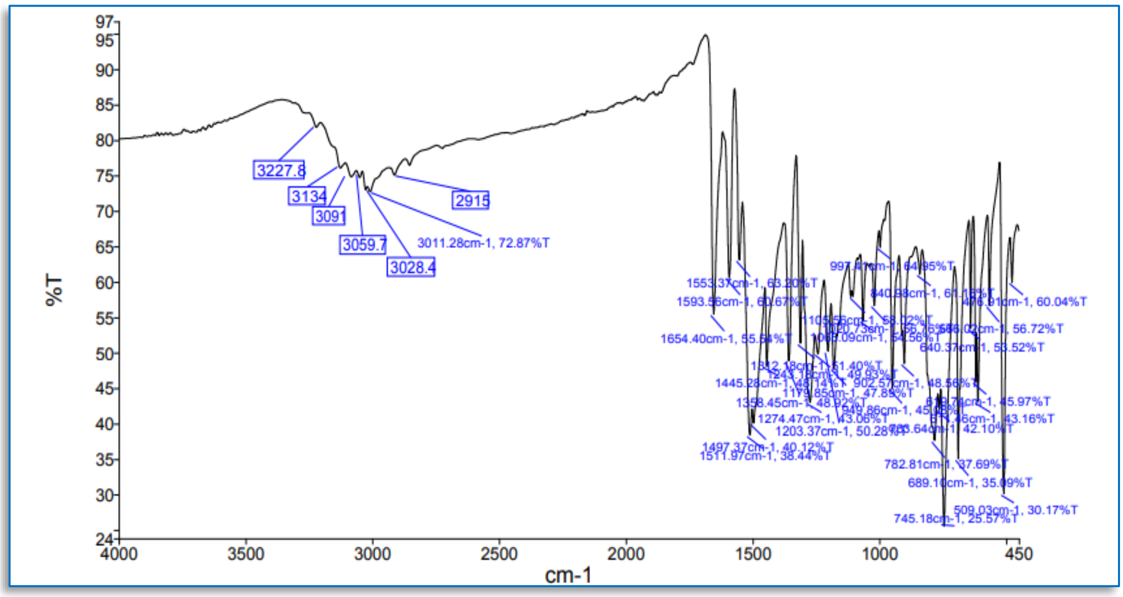

List of Peak Area/Height		
Peak Number	X (cm-1)	Y (%T)
1	3011.28	72.87
2	1654.40	55.54
3	1593.56	60.67
4	1553.37	63.20
5	1511.97	38.44
6	1497.37	40.12
7	1445.28	48.14
8	1358.45	48.92
9	1312.18	51.40
10	1274.47	43.06
11	1243.18	49.93
12	1203.37	50.28
13	1179.85	47.89
14	1105.56	58.02
15	1065.09	54.56
16	1020.73	56.76
17	997.41	64.95
18	949.86	45.08
19	902.57	48.56
20	840.98	61.16
21	782.81	37.69
22	763.64	42.10
23	745.18	25.57
24	689.10	35.09
25	640.37	53.52
26	619.74	45.97
27	611.46	43.16
28	566.02	56.72
29	509.03	30.17
30	476.91	60.04

Fig. S1. IR spectrum of **2a**

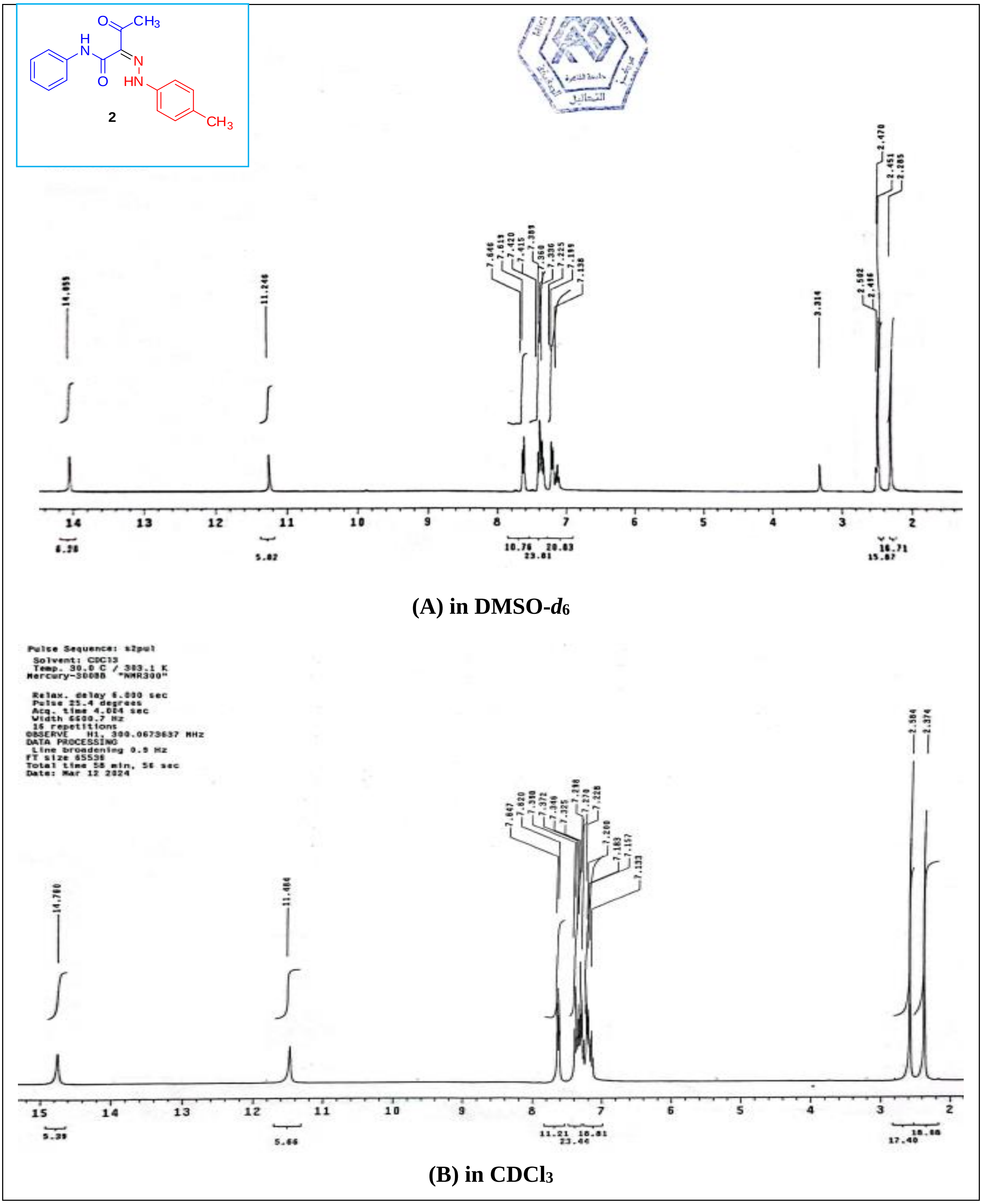

Fig. S2.^{1}H NMR spectra of **2a** in DMSO-d_6 and $CDCl_3$ at 300 MHz.

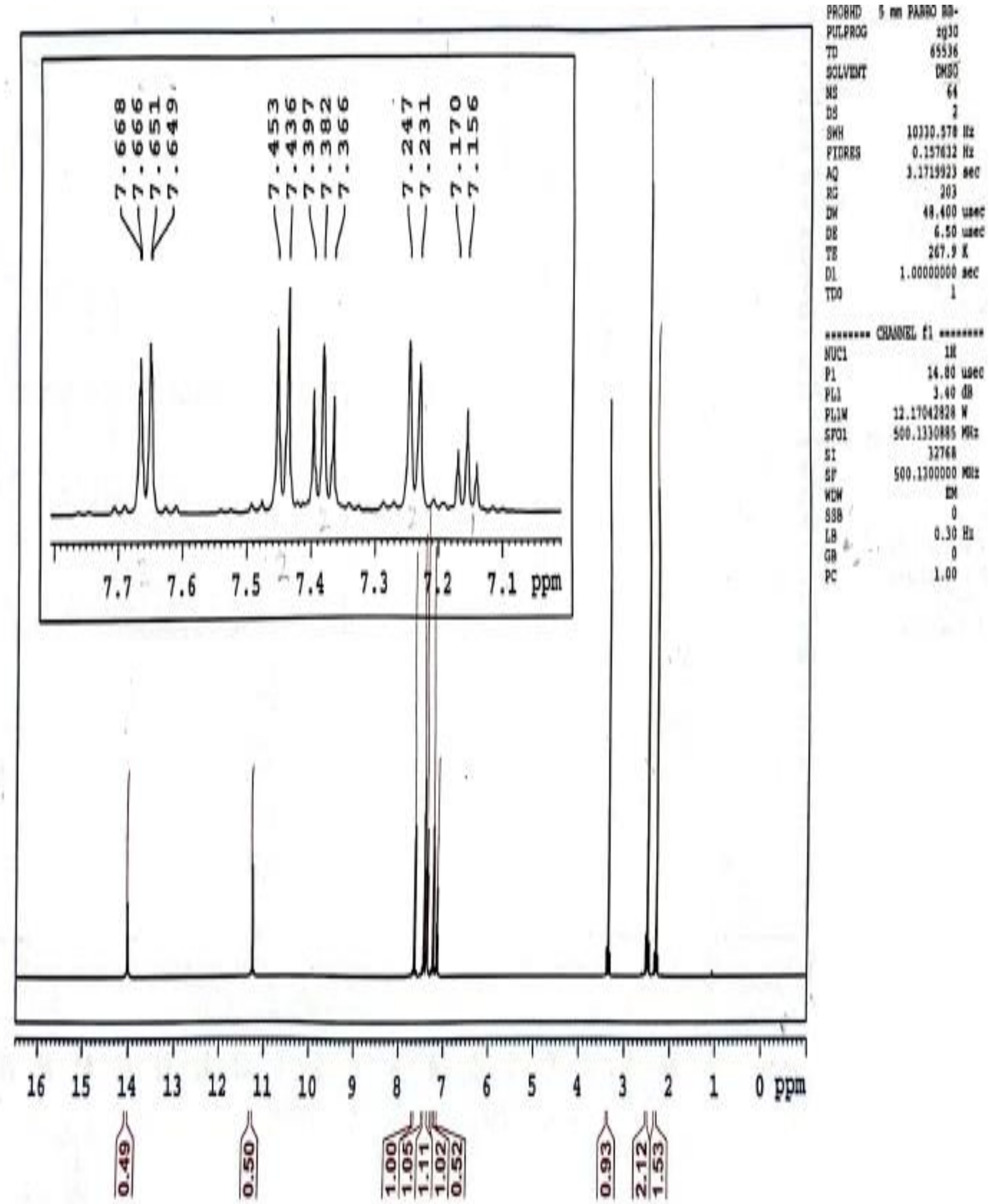

Fig. S3. ^{1}H NMR spectrum of hydrazone **2a** in DMSO-d_6 at 500 MHz

At 300 MHz

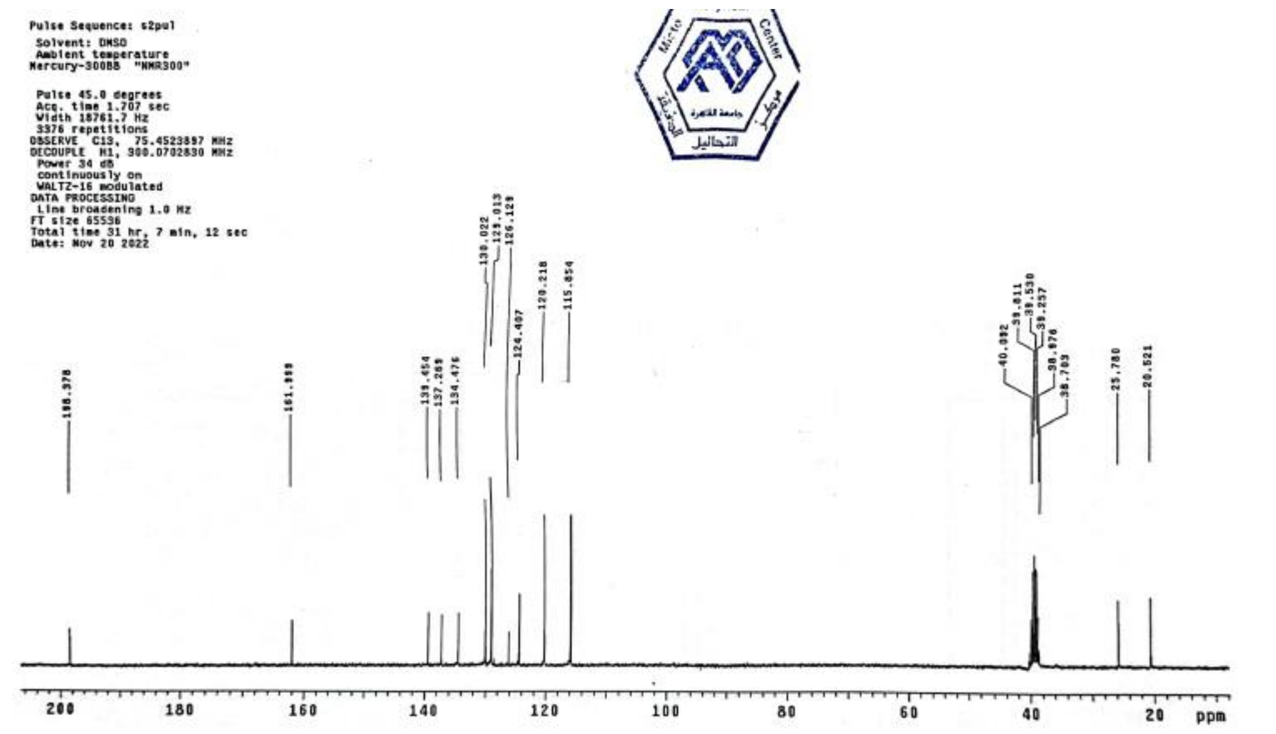

At 500 MHz

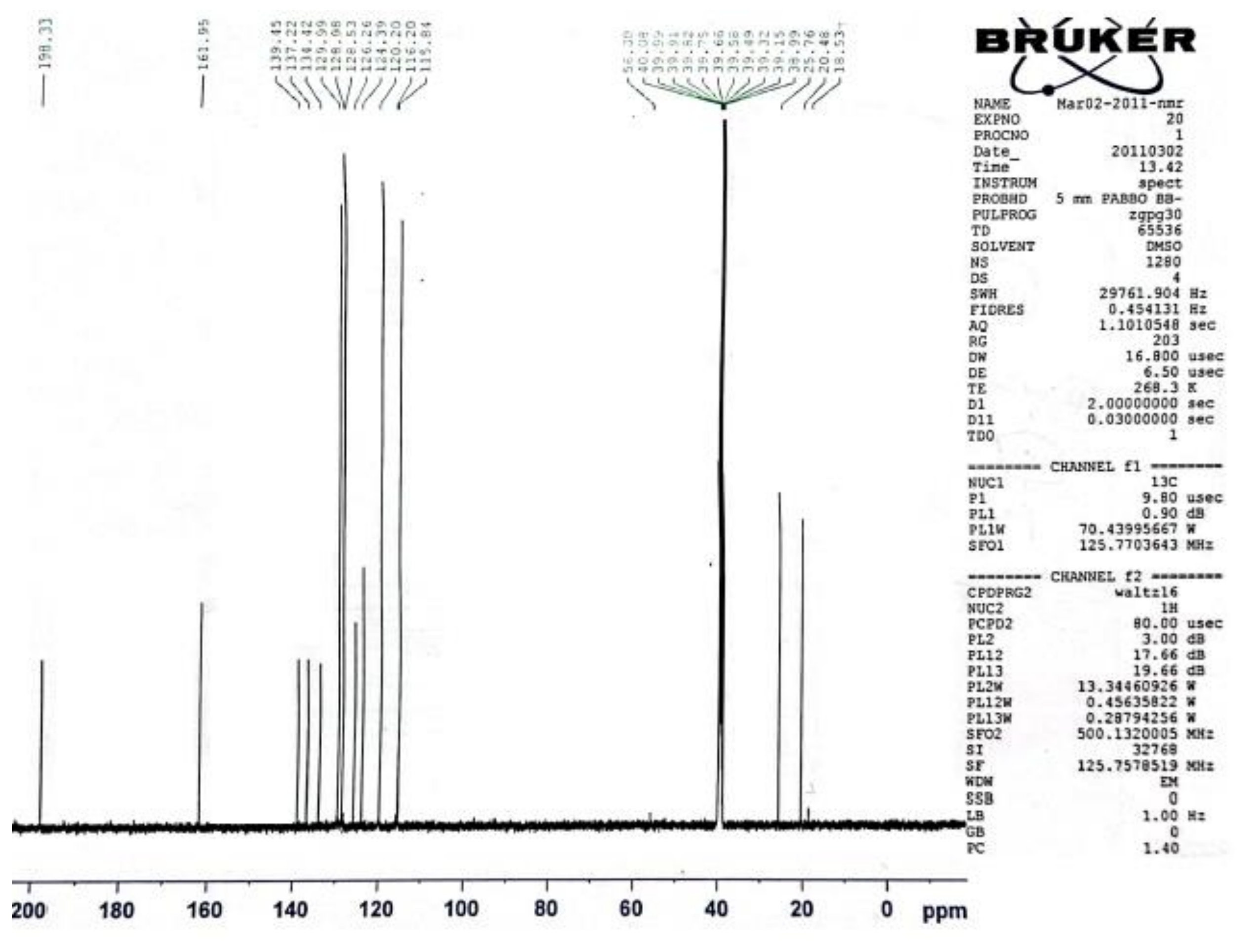

Fig. S4. ^{13}C NMR spectra of compound **2a in** DMSO-d_6

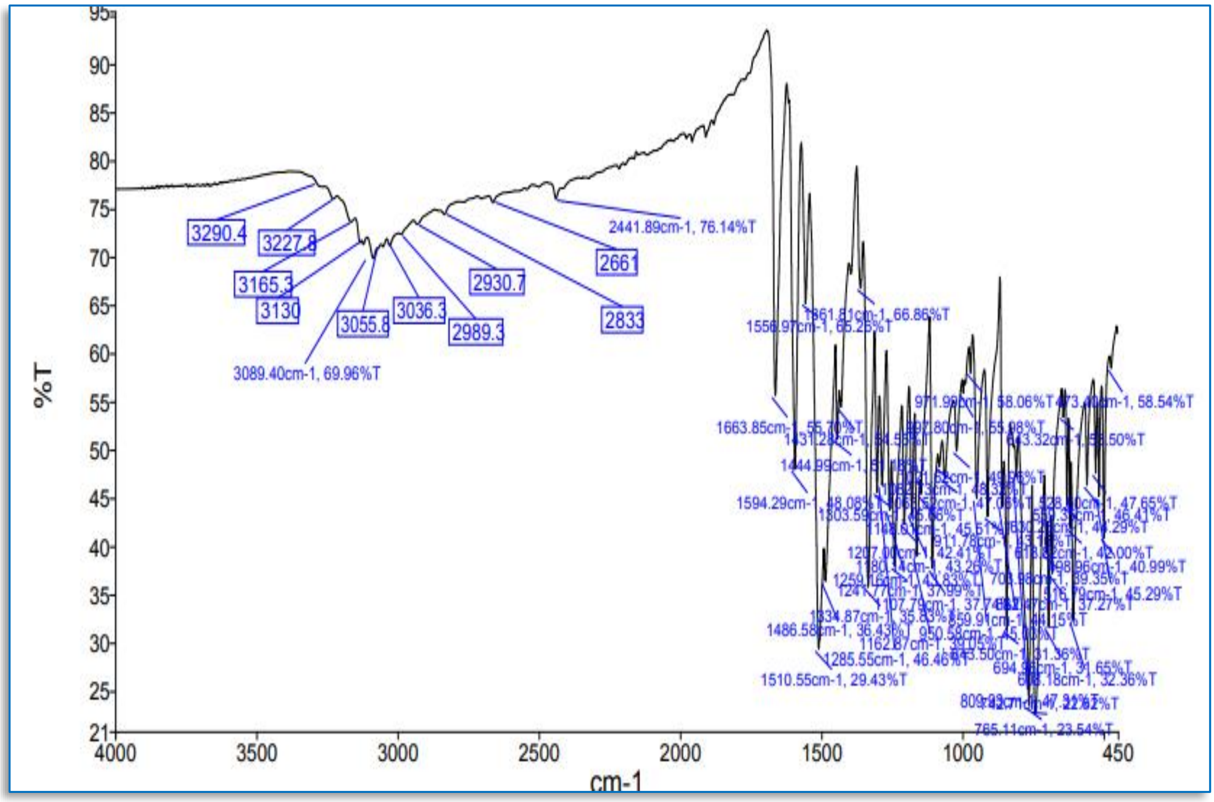

List of Peak Area/Height		
Peak Number	X (cm-1)	Y (%T)
1	3089.40	69.96
2	2441.89	76.14
3	1663.85	55.70
4	1594.29	48.08
5	1556.97	65.26
6	1510.55	29.43
7	1486.58	36.43
8	1444.99	51.18
9	1431.28	54.55
10	1361.81	66.86
11	1334.87	35.83
12	1303.59	45.66
13	1285.55	46.46
14	1259.16	43.83
15	1241.77	37.99
16	1207.00	42.41
17	1180.14	43.26
18	1162.87	39.05
19	1148.01	45.51
20	1107.79	37.74
21	1082.73	48.32
22	1063.52	47.06
23	1021.62	49.96
24	997.80	55.98
25	971.99	58.06
26	950.58	45.03
27	911.78	43.16
28	859.91	44.15
29	843.50	31.36
30	809.93	47.31
31	765.11	23.54
32	742.71	22.62

Fig. S5. IR spectrum of carboxamide **2b**.

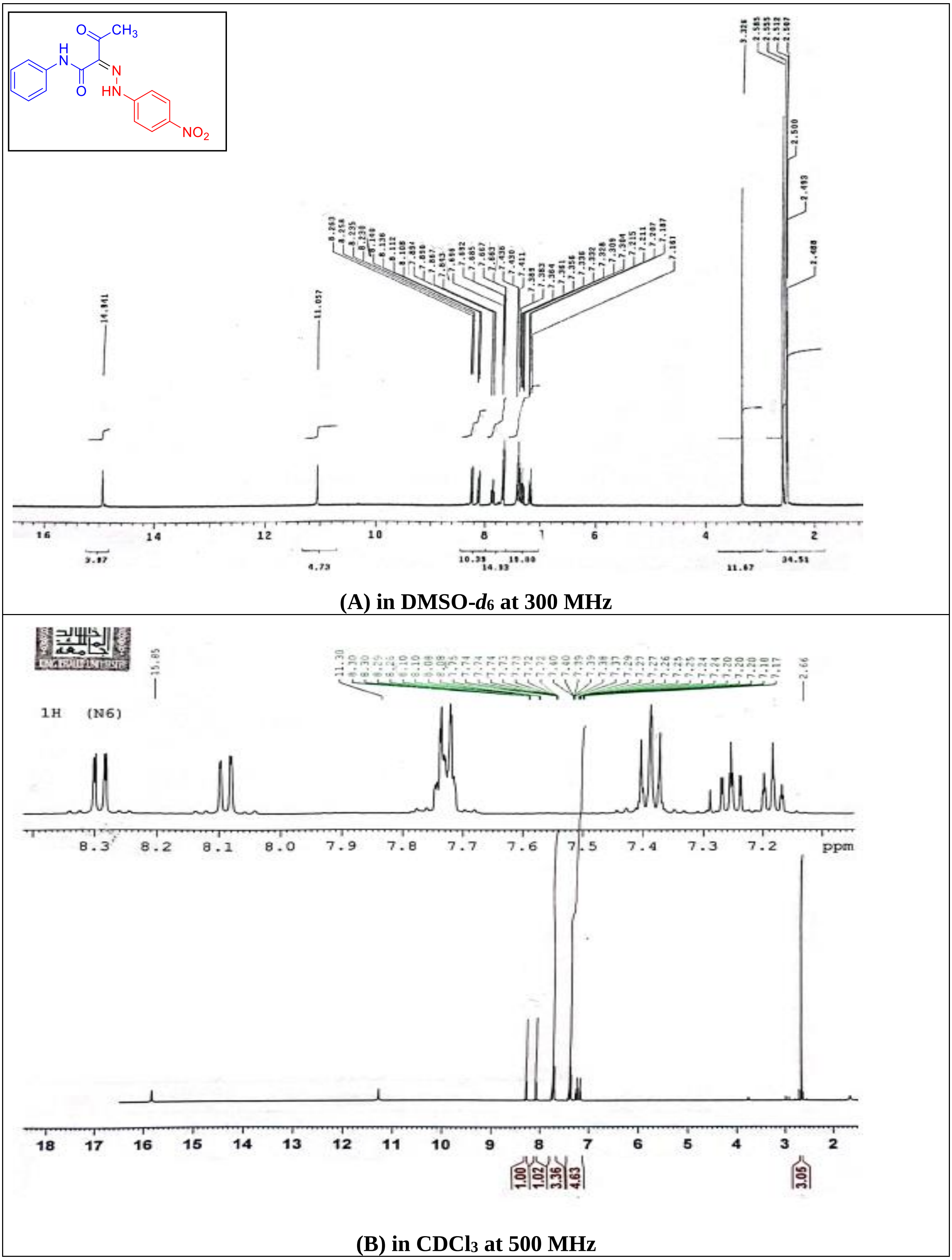

(A) in DMSO-d_6 at 300 MHz

(B) in $CDCl_3$ at 500 MHz

Fig. S6. ^{1}H NMR spectrum of compound **2b**.

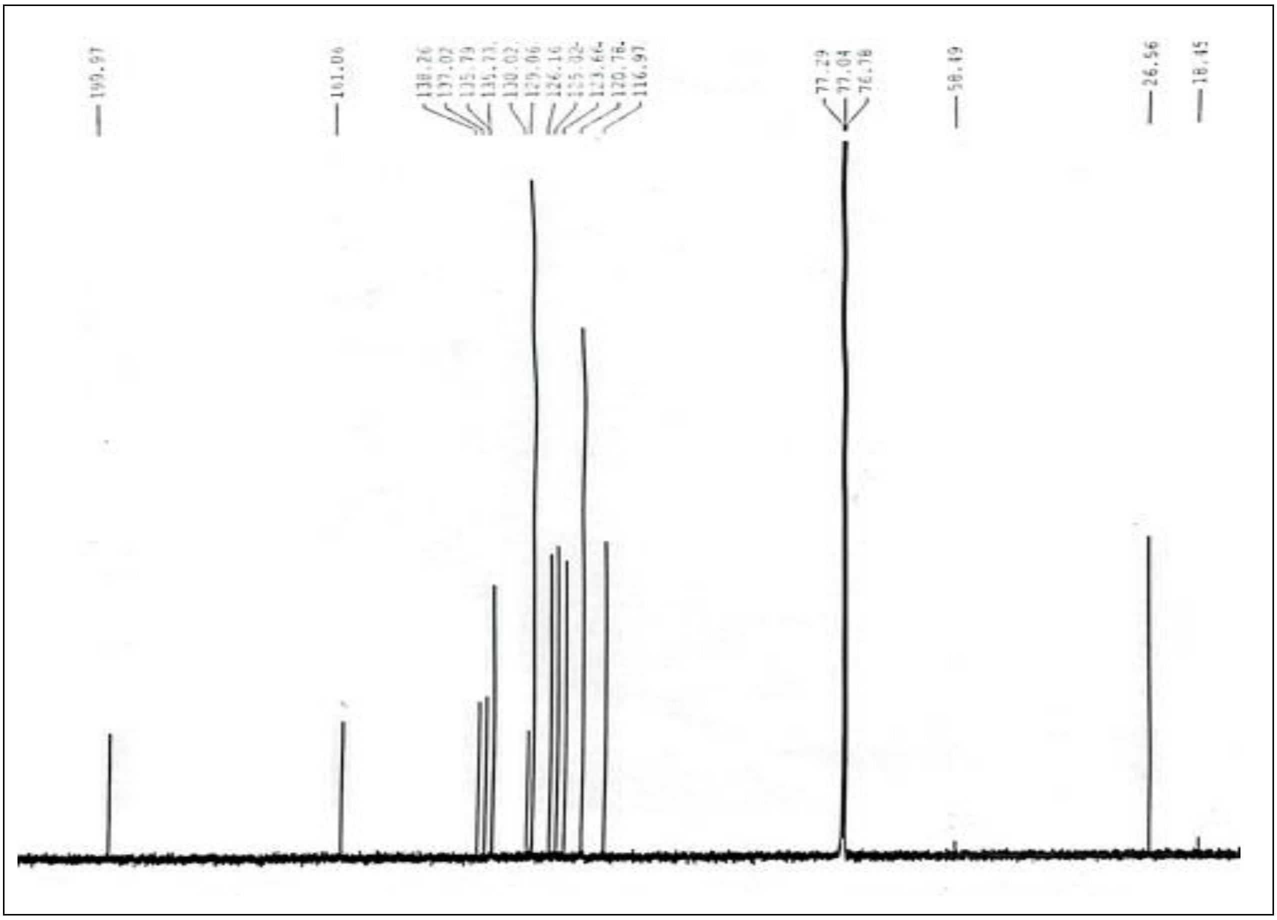

Fig. S7. ^{13}C NMR spectrum of compound **2b** in $CDCl_3$ at 500 MHz.

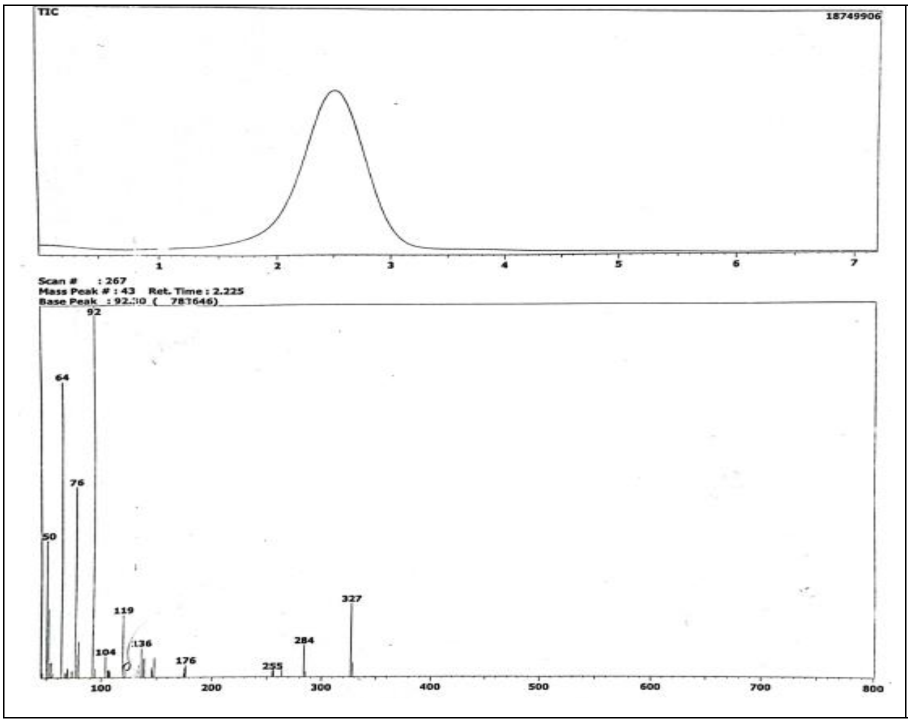

Data : NS15.D09
Scan # : 267
Mass Peak # : 43 Ret. Time : 2.225

Mass	R.Int	Mass	R.Int
46.30	1.29	121.25	1.88
50.50	36.98	130.45	1.29
52.00	18.75	132.40	1.99
53.25	4.01	133.90	4.97
54.65	4.22	134.75	1.55
56.45	1.17	136.10	7.78
63.95	79.63	137.50	4.91
67.05	1.43	138.85	5.17
68.40	1.18	145.40	2.69
69.55	2.44	146.40	1.87
73.45	1.78	147.95	5.29
76.50	51.12	174.60	1.02
78.05	6.17	175.40	2.53
79.55	9.81	176.30	3.17
92.30	100.00	255.30	1.53
94.30	2.51	256.10	1.98
103.60	5.60	263.85	2.43
105.40	1.99	283.65	8.40
106.35	1.90	284.95	1.41
107.15	1.88	326.85	19.57
108.05	1.53	328.20	3.69
119.30	16.78		

Fig. S8. Mass spectrum of carboxamide derivative **2b**.

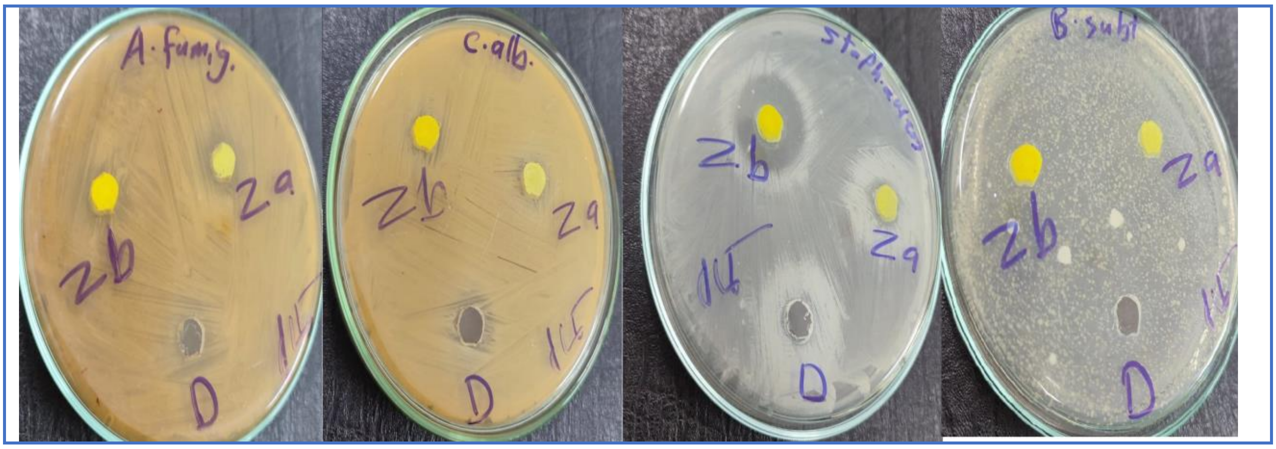

Fig. S9. Some Images of antimicrobial activity of 2a and **2b**

<u>CIF data</u>

data_solve2

_audit_creation_method 'SHELXL-2017/1'

_shelx_SHELXL_version_number '2017/1'

_chemical_name_systematic ?

_chemical_name_common ?

_chemical_melting_point ?

_chemical_formula_moiety ?

_chemical_formula_sum

'C17 H17 N3 O2'

_chemical_formula_weight 295.33

loop_

_atom_type_symbol

_atom_type_description

_atom_type_scat_dispersion_real

_atom_type_scat_dispersion_imag

_atom_type_scat_source

'C' 'C' 0.0181 0.0091

'International Tables Vol C Tables 4.2.6.8 and 6.1.1.4'

'H' 'H' 0.0000 0.0000

'International Tables Vol C Tables 4.2.6.8 and 6.1.1.4'

'N' 'N' 0.0311 0.0180

'International Tables Vol C Tables 4.2.6.8 and 6.1.1.4'

'O' 'O' 0.0492 0.0322

'International Tables Vol C Tables 4.2.6.8 and 6.1.1.4'

_space_group_crystal_system triclinic

_space_group_IT_number 2

_space_group_name_H-M_alt 'P -1'

_space_group_name_Hall '-P 1'

_shelx_space_group_comment

;

The symmetry employed for this shelxl refinement is uniquely defined

by the following loop, which should always be used as a source of

symmetry information in preference to the above space-group names.

They are only intended as comments.

;

loop_

_space_group_symop_operation_xyz

'x, y, z'

'-x, -y, -z'

_cell_length_a 8.0586(5)

_cell_length_b 12.0229(8)

_cell_length_c 16.9468(9)

_cell_angle_alpha 71.801(4)

_cell_angle_beta 83.300(4)

_cell_angle_gamma 84.316(4)

_cell_volume 1545.74(17)

_cell_formula_units_Z 4

_cell_measurement_temperature 296(2)

_cell_measurement_reflns_used 7903

_cell_measurement_theta_min 2.7561

_cell_measurement_theta_max 65.6749

_exptl_crystal_description Block

_exptl_crystal_colour yellow

_exptl_crystal_density_meas ?

_exptl_crystal_density_method ?

_exptl_crystal_density_diffrn 1.269

_exptl_crystal_F_000 624

_exptl_transmission_factor_min ?

_exptl_transmission_factor_max ?

_exptl_crystal_size_max 0.200

_exptl_crystal_size_mid 0.180

_exptl_crystal_size_min 0.110

_exptl_absorpt_coefficient_mu 0.690

_shelx_estimated_absorpt_T_min 0.874

_shelx_estimated_absorpt_T_max 0.928

_exptl_absorpt_correction_type Multi-Scan

_exptl_absorpt_process_details

;

SADABS-2016/2 - Bruker AXS area detector scaling and absorption correction

;

_exptl_absorpt_correction_T_min 0.64

_exptl_absorpt_correction_T_max 0.93

_diffrn_ambient_temperature 296(2)

_diffrn_radiation_wavelength 1.54178

_diffrn_radiation_type CuK\a

_diffrn_measurement_device_type 'Bruker APEX-II CCD'

_diffrn_measurement_method '\f and \w scans'

_diffrn_detector_area_resol_mean 8.3333

_diffrn_reflns_number 35411

_diffrn_reflns_av_unetI/netI 0.0438

_diffrn_reflns_av_R_equivalents 0.0681

_diffrn_reflns_limit_h_min -9

_diffrn_reflns_limit_h_max 9

_diffrn_reflns_limit_k_min -14

_diffrn_reflns_limit_k_max 14

_diffrn_reflns_limit_l_min -20

_diffrn_reflns_limit_l_max 19

_diffrn_reflns_theta_min 2.756

_diffrn_reflns_theta_max 66.661

_diffrn_reflns_theta_full 66.661

_diffrn_measured_fraction_theta_max 0.995

_diffrn_measured_fraction_theta_full 0.995

_diffrn_reflns_Laue_measured_fraction_max 0.995

_diffrn_reflns_Laue_measured_fraction_full 0.995

_diffrn_reflns_point_group_measured_fraction_max 0.995

_diffrn_reflns_point_group_measured_fraction_full 0.995

_reflns_number_total 5461

_reflns_number_gt 3664

_reflns_threshold_expression 'I > 2\s(I)'

_reflns_Friedel_coverage 0.000

_reflns_Friedel_fraction_max .

_reflns_Friedel_fraction_full .

_reflns_special_details

;

Reflections were merged by SHELXL according to the crystal

class for the calculation of statistics and refinement.

_reflns_Friedel_fraction is defined as the number of unique

Friedel pairs measured divided by the number that would be

possible theoretically, ignoring centric projections and

systematic absences.

;

_computing_data_collection ?

_computing_cell_refinement ?

_computing_data_reduction ?

_computing_structure_solution 'SHELXT 2014/5 (Sheldrick, 2014)'

_computing_structure_refinement 'SHELXL-2017/1 (Sheldrick, 2017)'

_computing_molecular_graphics ?

_computing_publication_material ?

_refine_special_details ?

_refine_ls_structure_factor_coef Fsqd

_refine_ls_matrix_type full

_refine_ls_weighting_scheme calc

_refine_ls_weighting_details

'w=1/[\s^2^(Fo^2^)+(0.0593P)^2^+0.5502P] where P=(Fo^2^+2Fc^2^)/3'

_atom_sites_solution_primary ?

_atom_sites_solution_secondary ?

_atom_sites_solution_hydrogens geom

_refine_ls_hydrogen_treatment mixed

_refine_ls_extinction_method none

_refine_ls_extinction_coef .

_refine_ls_number_reflns 5461

_refine_ls_number_parameters 405

_refine_ls_number_restraints 4

_refine_ls_R_factor_all 0.0831

_refine_ls_R_factor_gt 0.0525

_refine_ls_wR_factor_ref 0.1573

_refine_ls_wR_factor_gt 0.1317

_refine_ls_goodness_of_fit_ref 1.021

_refine_ls_restrained_S_all 1.020

_refine_ls_shift/su_max 0.000

_refine_ls_shift/su_mean 0.000

loop_

_atom_site_label

_atom_site_type_symbol

_atom_site_fract_x

_atom_site_fract_y

_atom_site_fract_z

_atom_site_U_iso_or_equiv

_atom_site_adp_type

_atom_site_occupancy

_atom_site_site_symmetry_order

_atom_site_calc_flag

_atom_site_refinement_flags_posn

_atom_site_refinement_flags_adp

_atom_site_refinement_flags_occupancy

_atom_site_disorder_assembly

_atom_site_disorder_group

O1B O -0.0345(2) 0.72401(17) 0.78814(10) 0.0780(5) Uani 1 1 d . . . . .

C1B C 0.0653(3) 0.71078(19) 0.62130(14) 0.0539(5) Uani 1 1 d . . . . .

N1B N 0.1351(2) 0.65087(16) 0.69727(11) 0.0583(5) Uani 1 1 d D . . . .

H1B H 0.2214(10) 0.5989(6) 0.69422(12) 0.070 Uiso 1 1 calc DR U . . .

C1A C 0.3882(3) 0.83880(18) 0.30701(13) 0.0516(5) Uani 1 1 d . . . . .

N1A N 0.4666(2) 0.78083(16) 0.38080(10) 0.0558(5) Uani 1 1 d D . . . .

H1A H 0.5269(7) 0.7143(7) 0.38112(10) 0.067 Uiso 1 1 calc DR U . . .

O1A O 0.4002(2) 0.90751(13) 0.45709(9) 0.0720(5) Uani 1 1 d . . . . .

O2B O 0.3745(2) 0.49023(15) 0.76342(11) 0.0760(5) Uani 1 1 d . . . . .

C2B C -0.0413(3) 0.8115(2) 0.60975(16) 0.0644(6) Uani 1 1 d . . . . .

H2B H -0.072648 0.842476 0.653595 0.077 Uiso 1 1 calc R U . . .

N2B N 0.1670(2) 0.59950(17) 0.91735(12) 0.0582(5) Uani 1 1 d . . . . .

C2A C 0.2834(3) 0.9394(2) 0.29620(14) 0.0624(6) Uani 1 1 d . . . . .

H2A H 0.263555 0.974947 0.338192 0.075 Uiso 1 1 calc R U . . .

N2A N 0.5260(2) 0.73689(15) 0.59791(10) 0.0499(4) Uani 1 1 d . . . . .

O2A O 0.6494(2) 0.58668(14) 0.45335(10) 0.0708(5) Uani 1 1 d . . . . .

C3A C 0.2078(3) 0.9872(2) 0.22249(16) 0.0711(7) Uani 1 1 d . . . . .

H3A H 0.137106 1.054880 0.215349 0.085 Uiso 1 1 calc R U . . .

N3A N 0.4510(2) 0.82967(15) 0.61299(11) 0.0545(5) Uani 1 1 d D . . . .

H3A1 H 0.4089(5) 0.8868(7) 0.5724(5) 0.065 Uiso 1 1 calc DR U . . .

C4A C 0.2362(4) 0.9357(2) 0.15985(16) 0.0759(7) Uani 1 1 d . . . . .

H4A H 0.185067 0.968176 0.110553 0.091 Uiso 1 1 calc R U . . .

N3B N 0.0450(2) 0.66827(18) 0.93801(12) 0.0622(5) Uani 1 1 d D . . . .

H3B1 H -0.0228(8) 0.7108(5) 0.8996(4) 0.075 Uiso 1 1 calc DR U . . .

C3B C -0.1004(3) 0.8654(2) 0.53272(16) 0.0710(7) Uani 1 1 d . . . . .

H3B H -0.171518 0.932968 0.524936 0.085 Uiso 1 1 calc R U . . .

C5A C 0.3407(4) 0.8360(2) 0.17080(15) 0.0774(8) Uani 1 1 d . . . . .

H5A H 0.360502 0.801012 0.128507 0.093 Uiso 1 1 calc R U . . .

C4B C -0.0555(3) 0.8206(2) 0.46751(17) 0.0756(7) Uani 1 1 d . . . . .

H4B H -0.095741 0.857515 0.415792 0.091 Uiso 1 1 calc R U . . .

C6A C 0.4169(3) 0.7869(2) 0.24376(14) 0.0634(6) Uani 1 1 d . . . . .

H6A H 0.487352 0.719162 0.250503 0.076 Uiso 1 1 calc R U . . .

C5B C 0.0491(3) 0.7208(2) 0.47935(16) 0.0753(7) Uani 1 1 d . . . . .

H5B H 0.079482 0.689996 0.435371 0.090 Uiso 1 1 calc R U . . .

C7A C 0.4627(3) 0.81243(19) 0.45087(13) 0.0513(5) Uani 1 1 d . . . . .

C6B C 0.1099(3) 0.6656(2) 0.55577(14) 0.0608(6) Uani 1 1 d . . . . .

H6B H 0.180886 0.598032 0.563068 0.073 Uiso 1 1 calc R U . . .

C8A C 0.5378(3) 0.72365(18) 0.52271(13) 0.0487(5) Uani 1 1 d . . . . .

C7B C 0.0887(3) 0.6618(2) 0.77347(14) 0.0548(5) Uani 1 1 d . . . . .

C9A C 0.6272(3) 0.61328(19) 0.51837(14) 0.0560(6) Uani 1 1 d . . . . .

C8B C 0.1928(3) 0.59278(19) 0.84075(14) 0.0536(5) Uani 1 1 d . . . . .

C10A C 0.6943(3) 0.5309(2) 0.59660(15) 0.0721(7) Uani 1 1 d . . . . .

H10A H 0.746383 0.461434 0.585391 0.108 Uiso 1 1 calc R U . . .

H10B H 0.603918 0.510000 0.639554 0.108 Uiso 1 1 calc R U . . .

H10C H 0.775278 0.568904 0.614690 0.108 Uiso 1 1 calc R U . . .

C9B C 0.3353(3) 0.5103(2) 0.83004(16) 0.0615(6) Uani 1 1 d . . . . .

C11A C 0.4369(3) 0.83892(18) 0.69444(13) 0.0513(5) Uani 1 1 d . . . . .

C10B C 0.4335(3) 0.4489(3) 0.90263(19) 0.0890(9) Uani 1 1 d . . . . .

H10D H 0.518027 0.395478 0.887158 0.134 Uiso 1 1 calc R U . . .

H10E H 0.359956 0.406052 0.948394 0.134 Uiso 1 1 calc R U . . .

H10F H 0.485717 0.505594 0.919022 0.134 Uiso 1 1 calc R U . . .

C12A C 0.5396(3) 0.7710(2) 0.75376(14) 0.0587(6) Uani 1 1 d . . . . .

H12A H 0.621062 0.717474 0.740826 0.070 Uiso 1 1 calc R U . . .

C11B C 0.0190(3) 0.6762(2) 1.01955(14) 0.0607(6) Uani 1 1 d . . . . .

C13A C 0.5194(3) 0.7838(2) 0.83226(15) 0.0673(7) Uani 1 1 d . . . . .

H13A H 0.588667 0.737979 0.872031 0.081 Uiso 1 1 calc R U . . .

C12B C 0.1056(3) 0.6038(2) 1.08344(16) 0.0679(7) Uani 1 1 d . . . . .

H12B H 0.185607 0.547444 1.073247 0.082 Uiso 1 1 calc R U . . .

C14A C 0.4006(3) 0.8619(2) 0.85414(14) 0.0661(7) Uani 1 1 d . . . . .

C13B C 0.0742(3) 0.6143(3) 1.16322(16) 0.0752(7) Uani 1 1 d . . . . .

H13B H 0.134467 0.565000 1.205763 0.090 Uiso 1 1 calc R U . . .

C15A C 0.3000(3) 0.9292(2) 0.79371(16) 0.0725(7) Uani 1 1 d . . . . .

H15A H 0.218443 0.982636 0.806763 0.087 Uiso 1 1 calc R U . . .

C14B C -0.0443(4) 0.6962(3) 1.18076(16) 0.0772(8) Uani 1 1 d . . . . .

C16A C 0.3180(3) 0.9185(2) 0.71398(15) 0.0653(6) Uani 1 1 d . . . . .

H16A H 0.249937 0.965110 0.673885 0.078 Uiso 1 1 calc R U . . .

C15B C -0.1310(4) 0.7684(3) 1.11559(17) 0.0818(8) Uani 1 1 d . . . . .

H15B H -0.211617 0.824431 1.125768 0.098 Uiso 1 1 calc R U . . .

C17A C 0.3813(4) 0.8758(3) 0.94049(16) 0.0949(10) Uani 1 1 d . . . . .

H17A H 0.400749 0.800695 0.981032 0.142 Uiso 1 1 calc R U . . .

H17B H 0.270001 0.907308 0.951617 0.142 Uiso 1 1 calc R U . . .

H17C H 0.460998 0.928243 0.943640 0.142 Uiso 1 1 calc R U . . .

C17B C -0.0774(4) 0.7068(3) 1.26684(18) 0.1089(11) Uani 1 1 d . . . . .

H17D H -0.095076 0.630726 1.306032 0.163 Uiso 1 1 calc R U . . .

H17E H -0.175437 0.758271 1.269554 0.163 Uiso 1 1 calc R U . . .

H17F H 0.016897 0.738084 1.280246 0.163 Uiso 1 1 calc R U . . .

C16B C -0.1007(3) 0.7591(2) 1.03618(16) 0.0749(7) Uani 1 1 d . . . . .

H16B H -0.160635 0.808538 0.993542 0.090 Uiso 1 1 calc R U . . .

loop_

_atom_site_aniso_label

_atom_site_aniso_U_11

_atom_site_aniso_U_22

_atom_site_aniso_U_33

_atom_site_aniso_U_23

_atom_site_aniso_U_13

_atom_site_aniso_U_12

O1B 0.0719(11) 0.1002(14) 0.0619(10) -0.0334(10) -0.0078(8) 0.0252(10)

C1B 0.0523(12) 0.0574(13) 0.0548(13) -0.0226(11) -0.0010(10) -0.0029(10)

N1B 0.0588(11) 0.0618(12) 0.0559(11) -0.0243(9) -0.0039(9) 0.0075(9)

C1A 0.0627(13) 0.0492(12) 0.0455(12) -0.0193(10) 0.0007(10) -0.0067(10)

N1A 0.0718(12) 0.0532(11) 0.0469(10) -0.0248(8) -0.0057(9) 0.0071(9)

O1A 0.1159(14) 0.0532(9) 0.0528(9) -0.0272(8) -0.0169(9) 0.0141(9)

O2B 0.0752(11) 0.0788(12) 0.0758(12) -0.0333(10) -0.0038(9) 0.0148(9)

C2B 0.0694(15) 0.0575(14) 0.0690(16) -0.0283(12) -0.0037(12) 0.0092(12)

N2B 0.0527(11) 0.0646(12) 0.0610(12) -0.0244(10) -0.0027(9) -0.0070(9)

C2A 0.0806(16) 0.0576(14) 0.0549(14) -0.0277(11) -0.0066(12) 0.0029(12)

N2A 0.0553(10) 0.0499(10) 0.0489(10) -0.0217(8) -0.0023(8) -0.0046(8)

O2A 0.0864(12) 0.0667(11) 0.0643(10) -0.0345(9) -0.0037(9) 0.0158(9)

C3A 0.0884(18) 0.0598(15) 0.0667(16) -0.0216(13) -0.0168(14) 0.0061(13)

N3A 0.0752(12) 0.0482(10) 0.0433(10) -0.0201(8) -0.0062(9) 0.0025(9)

C4A 0.100(2) 0.0724(17) 0.0569(15) -0.0173(13) -0.0193(14) -0.0076(15)

N3B 0.0612(12) 0.0771(13) 0.0523(11) -0.0274(10) -0.0027(9) -0.0002(10)

C3B 0.0711(16) 0.0649(16) 0.0743(17) -0.0224(13) -0.0078(13) 0.0131(13)

C5A 0.114(2) 0.0767(18) 0.0504(14) -0.0318(13) -0.0080(14) -0.0054(16)

C4B 0.0759(17) 0.0831(19) 0.0651(16) -0.0214(14) -0.0099(13) 0.0077(15)

C6A 0.0854(17) 0.0597(14) 0.0507(13) -0.0277(11) -0.0023(12) 0.0006(12)

C5B 0.0833(18) 0.0857(19) 0.0622(16) -0.0349(14) -0.0069(13) 0.0106(15)

C7A 0.0632(13) 0.0479(12) 0.0472(12) -0.0223(10) -0.0010(10) -0.0031(10)

C6B 0.0624(14) 0.0639(14) 0.0595(14) -0.0286(12) -0.0033(11) 0.0092(11)

C8A 0.0539(12) 0.0475(12) 0.0481(12) -0.0208(10) 0.0003(9) -0.0041(9)

C7B 0.0506(12) 0.0627(14) 0.0555(13) -0.0262(11) -0.0007(10) -0.0019(11)

C9A 0.0577(13) 0.0548(13) 0.0588(14) -0.0250(11) 0.0008(11) -0.0003(11)

C8B 0.0470(12) 0.0579(13) 0.0604(14) -0.0252(11) -0.0009(10) -0.0061(10)

C10A 0.0841(17) 0.0617(15) 0.0688(16) -0.0227(13) -0.0097(13) 0.0156(13)

C9B 0.0554(13) 0.0638(15) 0.0692(16) -0.0260(12) -0.0050(12) -0.0046(11)

C11A 0.0651(13) 0.0499(12) 0.0436(12) -0.0200(10) -0.0040(10) -0.0084(10)

C10B 0.0758(18) 0.096(2) 0.101(2) -0.0364(17) -0.0301(16) 0.0186(16)

C12A 0.0677(14) 0.0575(14) 0.0548(13) -0.0224(11) -0.0080(11) -0.0021(11)

C11B 0.0590(14) 0.0756(16) 0.0520(13) -0.0242(12) 0.0022(11) -0.0182(12)

C13A 0.0855(17) 0.0674(15) 0.0512(14) -0.0161(12) -0.0153(12) -0.0118(14)

C12B 0.0604(14) 0.0802(17) 0.0677(16) -0.0266(13) -0.0074(12) -0.0107(13)

C14A 0.0841(17) 0.0750(16) 0.0458(13) -0.0254(12) 0.0004(12) -0.0205(14)

C13B 0.0753(17) 0.096(2) 0.0583(16) -0.0237(14) -0.0093(13) -0.0196(15)

C15A 0.0825(17) 0.0784(17) 0.0666(16) -0.0416(14) 0.0028(14) 0.0011(14)

C14B 0.0811(18) 0.100(2) 0.0587(16) -0.0324(15) 0.0057(14) -0.0301(16)

C16A 0.0796(16) 0.0655(15) 0.0562(14) -0.0286(12) -0.0119(12) 0.0101(13)

C15B 0.0821(19) 0.094(2) 0.0732(18) -0.0363(16) 0.0071(15) -0.0073(16)

C17A 0.125(3) 0.115(2) 0.0548(16) -0.0387(16) 0.0014(16) -0.027(2)

C17B 0.121(3) 0.151(3) 0.0643(18) -0.045(2) 0.0101(17) -0.033(2)

C16B 0.0785(17) 0.0847(19) 0.0621(16) -0.0286(14) 0.0009(13) 0.0034(15)

_geom_special_details

;

All esds (except the esd in the dihedral angle between two l.s. planes)
are estimated using the full covariance matrix. The cell esds are taken
into account individually in the estimation of esds in distances, angles
and torsion angles; correlations between esds in cell parameters are only
used when they are defined by crystal symmetry. An approximate (isotropic)
treatment of cell esds is used for estimating esds involving l.s. planes.

;

loop_
 _geom_bond_atom_site_label_1
 _geom_bond_atom_site_label_2
 _geom_bond_distance
 _geom_bond_site_symmetry_2
 _geom_bond_publ_flag
O1B C7B 1.231(3) . ?
C1B C6B 1.379(3) . ?
C1B C2B 1.388(3) . ?
C1B N1B 1.415(3) . ?
N1B C7B 1.345(3) . ?
N1B H1B 0.895(10) . ?
C1A C2A 1.380(3) . ?
C1A C6A 1.386(3) . ?
C1A N1A 1.415(3) . ?
N1A C7A 1.351(3) . ?
N1A H1A 0.893(10) . ?
O1A C7A 1.233(2) . ?
O2B C9B 1.226(3) . ?
C2B C3B 1.381(3) . ?
C2B H2B 0.9300 . ?
N2B N3B 1.308(2) . ?
N2B C8B 1.317(3) . ?
C2A C3A 1.386(3) . ?
C2A H2A 0.9300 . ?
N2A N3A 1.297(2) . ?
N2A C8A 1.324(3) . ?

O2A C9A 1.229(2) . ?

C3A C4A 1.374(3) . ?

C3A H3A 0.9300 . ?

N3A C11A 1.410(3) . ?

N3A H3A1 0.879(10) . ?

C4A C5A 1.370(4) . ?

C4A H4A 0.9300 . ?

N3B C11B 1.405(3) . ?

N3B H3B1 0.896(10) . ?

C3B C4B 1.372(3) . ?

C3B H3B 0.9300 . ?

C5A C6A 1.380(3) . ?

C5A H5A 0.9300 . ?

C4B C5B 1.370(4) . ?

C4B H4B 0.9300 . ?

C6A H6A 0.9300 . ?

C5B C6B 1.380(3) . ?

C5B H5B 0.9300 . ?

C7A C8A 1.490(3) . ?

C6B H6B 0.9300 . ?

C8A C9A 1.464(3) . ?

C7B C8B 1.479(3) . ?

C9A C10A 1.507(3) . ?

C8B C9B 1.474(3) . ?

C10A H10A 0.9600 . ?

C10A H10B 0.9600 . ?

C10A H10C 0.9600 . ?

C9B C10B 1.493(3) . ?

C11A C16A 1.373(3) . ?

C11A C12A 1.382(3) . ?

C10B H10D 0.9600 . ?

C10B H10E 0.9600 . ?

C10B H10F 0.9600 . ?

C12A C13A 1.376(3) . ?

C12A H12A 0.9300 . ?

C11B C12B 1.373(3) . ?

C11B C16B 1.387(3) . ?

C13A C14A 1.375(3) . ?

C13A H13A 0.9300 . ?

C12B C13B 1.389(3) . ?

C12B H12B 0.9300 . ?

C14A C15A 1.381(4) . ?

C14A C17A 1.513(3) . ?

C13B C14B 1.379(4) . ?

C13B H13B 0.9300 . ?

C15A C16A 1.386(3) . ?

C15A H15A 0.9300 . ?

C14B C15B 1.387(4) . ?

C14B C17B 1.494(4) . ?

C16A H16A 0.9300 . ?

C15B C16B 1.377(3) . ?

C15B H15B 0.9300 . ?

C17A H17A 0.9600 . ?

C17A H17B 0.9600 . ?

C17A H17C 0.9600 . ?

C17B H17D 0.9600 . ?

C17B H17E 0.9600 . ?

C17B H17F 0.9600 . ?

C16B H16B 0.9300 . ?

loop_
 _geom_angle_atom_site_label_1
 _geom_angle_atom_site_label_2
 _geom_angle_atom_site_label_3
 _geom_angle
 _geom_angle_site_symmetry_1
 _geom_angle_site_symmetry_3
 _geom_angle_publ_flag
C6B C1B C2B 119.5(2) . . ?
C6B C1B N1B 116.79(19) . . ?
C2B C1B N1B 123.7(2) . . ?
C7B N1B C1B 128.40(19) . . ?
C7B N1B H1B 115.8 . . ?
C1B N1B H1B 115.8 . . ?
C2A C1A C6A 119.5(2) . . ?
C2A C1A N1A 124.16(19) . . ?
C6A C1A N1A 116.35(19) . . ?
C7A N1A C1A 128.84(18) . . ?
C7A N1A H1A 115.6 . . ?
C1A N1A H1A 115.6 . . ?
C3B C2B C1B 119.5(2) . . ?
C3B C2B H2B 120.2 . . ?
C1B C2B H2B 120.2 . . ?
N3B N2B C8B 121.00(19) . . ?
C1A C2A C3A 119.7(2) . . ?
C1A C2A H2A 120.1 . . ?
C3A C2A H2A 120.1 . . ?

N3A N2A C8A 121.76(18) . . ?

C4A C3A C2A 120.8(2) . . ?

C4A C3A H3A 119.6 . . ?

C2A C3A H3A 119.6 . . ?

N2A N3A C11A 120.02(18) . . ?

N2A N3A H3A1 120.0 . . ?

C11A N3A H3A1 120.0 . . ?

C5A C4A C3A 119.3(2) . . ?

C5A C4A H4A 120.4 . . ?

C3A C4A H4A 120.4 . . ?

N2B N3B C11B 120.9(2) . . ?

N2B N3B H3B1 119.6 . . ?

C11B N3B H3B1 119.6 . . ?

C4B C3B C2B 120.9(2) . . ?

C4B C3B H3B 119.5 . . ?

C2B C3B H3B 119.5 . . ?

C4A C5A C6A 120.9(2) . . ?

C4A C5A H5A 119.6 . . ?

C6A C5A H5A 119.6 . . ?

C5B C4B C3B 119.3(2) . . ?

C5B C4B H4B 120.3 . . ?

C3B C4B H4B 120.3 . . ?

C5A C6A C1A 119.9(2) . . ?

C5A C6A H6A 120.0 . . ?

C1A C6A H6A 120.0 . . ?

C4B C5B C6B 120.8(2) . . ?

C4B C5B H5B 119.6 . . ?

C6B C5B H5B 119.6 . . ?

O1A C7A N1A 123.5(2) . . ?

O1A C7A C8A 120.74(18) . . ?

N1A C7A C8A 115.76(18) . . ?

C1B C6B C5B 120.0(2) . . ?

C1B C6B H6B 120.0 . . ?

C5B C6B H6B 120.0 . . ?

N2A C8A C9A 113.50(19) . . ?

N2A C8A C7A 122.35(18) . . ?

C9A C8A C7A 124.13(18) . . ?

O1B C7B N1B 123.0(2) . . ?

O1B C7B C8B 120.8(2) . . ?

N1B C7B C8B 116.23(19) . . ?

O2A C9A C8A 122.2(2) . . ?

O2A C9A C10A 119.6(2) . . ?

C8A C9A C10A 118.24(19) . . ?

N2B C8B C9B 112.8(2) . . ?

N2B C8B C7B 122.75(19) . . ?

C9B C8B C7B 124.4(2) . . ?

C9A C10A H10A 109.5 . . ?

C9A C10A H10B 109.5 . . ?

H10A C10A H10B 109.5 . . ?

C9A C10A H10C 109.5 . . ?

H10A C10A H10C 109.5 . . ?

H10B C10A H10C 109.5 . . ?

O2B C9B C8B 121.8(2) . . ?

O2B C9B C10B 119.2(2) . . ?

C8B C9B C10B 118.9(2) . . ?

C16A C11A C12A 120.0(2) . . ?

C16A C11A N3A 118.24(19) . . ?

C12A C11A N3A 121.7(2) . . ?

C9B C10B H10D 109.5 . . ?

C9B C10B H10E 109.5 . . ?

H10D C10B H10E 109.5 . . ?

C9B C10B H10F 109.5 . . ?

H10D C10B H10F 109.5 . . ?

H10E C10B H10F 109.5 . . ?

C13A C12A C11A 119.0(2) . . ?

C13A C12A H12A 120.5 . . ?

C11A C12A H12A 120.5 . . ?

C12B C11B C16B 119.0(2) . . ?

C12B C11B N3B 122.4(2) . . ?

C16B C11B N3B 118.6(2) . . ?

C14A C13A C12A 122.5(2) . . ?

C14A C13A H13A 118.8 . . ?

C12A C13A H13A 118.8 . . ?

C11B C12B C13B 120.2(3) . . ?

C11B C12B H12B 119.9 . . ?

C13B C12B H12B 119.9 . . ?

C13A C14A C15A 117.5(2) . . ?

C13A C14A C17A 121.8(2) . . ?

C15A C14A C17A 120.7(3) . . ?

C14B C13B C12B 121.5(3) . . ?

C14B C13B H13B 119.3 . . ?

C12B C13B H13B 119.3 . . ?

C14A C15A C16A 121.3(2) . . ?

C14A C15A H15A 119.4 . . ?

C16A C15A H15A 119.4 . . ?

C13B C14B C15B 117.5(2) . . ?

C13B C14B C17B 121.1(3) . . ?

C15B C14B C17B 121.4(3) . . ?

C11A C16A C15A 119.7(2) . . ?

C11A C16A H16A 120.1 . . ?

C15A C16A H16A 120.1 . . ?

C16B C15B C14B 121.6(3) . . ?

C16B C15B H15B 119.2 . . ?

C14B C15B H15B 119.2 . . ?

C14A C17A H17A 109.5 . . ?

C14A C17A H17B 109.5 . . ?

H17A C17A H17B 109.5 . . ?

C14A C17A H17C 109.5 . . ?

H17A C17A H17C 109.5 . . ?

H17B C17A H17C 109.5 . . ?

C14B C17B H17D 109.5 . . ?

C14B C17B H17E 109.5 . . ?

H17D C17B H17E 109.5 . . ?

C14B C17B H17F 109.5 . . ?

H17D C17B H17F 109.5 . . ?

H17E C17B H17F 109.5 . . ?

C15B C16B C11B 120.2(3) . . ?

C15B C16B H16B 119.9 . . ?

C11B C16B H16B 119.9 . . ?

loop_

_geom_torsion_atom_site_label_1

_geom_torsion_atom_site_label_2

_geom_torsion_atom_site_label_3

_geom_torsion_atom_site_label_4

_geom_torsion

_geom_torsion_site_symmetry_1

_geom_torsion_site_symmetry_2

_geom_torsion_site_symmetry_3

_geom_torsion_site_symmetry_4

_geom_torsion_publ_flag

C6B C1B N1B C7B -165.8(2) . . . . ?

C2B C1B N1B C7B 15.2(4) . . . . ?

C2A C1A N1A C7A -3.4(4) . . . . ?

C6A C1A N1A C7A 178.8(2) . . . . ?

C6B C1B C2B C3B -0.3(4) . . . . ?

N1B C1B C2B C3B 178.6(2) . . . . ?

C6A C1A C2A C3A 0.1(4) . . . . ?

N1A C1A C2A C3A -177.7(2) . . . . ?

C1A C2A C3A C4A -0.1(4) . . . . ?

C8A N2A N3A C11A 178.19(19) . . . . ?

C2A C3A C4A C5A 0.0(4) . . . . ?

C8B N2B N3B C11B -179.3(2) . . . . ?

C1B C2B C3B C4B 0.2(4) . . . . ?

C3A C4A C5A C6A 0.2(4) . . . . ?

C2B C3B C4B C5B 0.1(4) . . . . ?

C4A C5A C6A C1A -0.1(4) . . . . ?

C2A C1A C6A C5A 0.0(4) . . . . ?

N1A C1A C6A C5A 178.0(2) . . . . ?

C3B C4B C5B C6B -0.2(4) . . . . ?

C1A N1A C7A O1A -7.9(4) . . . . ?

C1A N1A C7A C8A 171.33(19) . . . . ?

C2B C1B C6B C5B 0.2(4) . . . . ?

N1B C1B C6B C5B -178.8(2) . . . . ?

C4B C5B C6B C1B 0.1(4) . . . . ?

N3A N2A C8A C9A 179.89(18) . . . . ?

N3A N2A C8A C7A -1.5(3) . . . . ?

O1A C7A C8A N2A 7.1(3) . . . . ?

N1A C7A C8A N2A -172.16(19) . . . . ?

O1A C7A C8A C9A -174.4(2) . . . . ?

N1A C7A C8A C9A 6.3(3) . . . . ?

C1B N1B C7B O1B 4.9(4) . . . . ?

C1B N1B C7B C8B -176.1(2) . . . . ?

N2A C8A C9A O2A 179.9(2) . . . . ?

C7A C8A C9A O2A 1.3(3) . . . . ?

N2A C8A C9A C10A -0.5(3) . . . . ?

C7A C8A C9A C10A -179.1(2) . . . . ?

N3B N2B C8B C9B -179.71(19) . . . . ?

N3B N2B C8B C7B 0.5(3) . . . . ?

O1B C7B C8B N2B -4.5(3) . . . . ?

N1B C7B C8B N2B 176.5(2) . . . . ?

O1B C7B C8B C9B 175.8(2) . . . . ?

N1B C7B C8B C9B -3.2(3) . . . . ?

N2B C8B C9B O2B 177.8(2) . . . . ?

C7B C8B C9B O2B -2.4(4) . . . . ?

N2B C8B C9B C10B -1.7(3) . . . . ?

C7B C8B C9B C10B 178.1(2) . . . . ?

N2A N3A C11A C16A -161.2(2) . . . . ?

N2A N3A C11A C12A 19.2(3) . . . . ?

C16A C11A C12A C13A 0.6(3) . . . . ?

N3A C11A C12A C13A -179.8(2) . . . . ?

N2B N3B C11B C12B -7.7(3) . . . . ?

N2B N3B C11B C16B 173.4(2) . . . . ?

C11A C12A C13A C14A 0.0(4) . . . . ?

C16B C11B C12B C13B -0.5(4) . . . . ?

N3B C11B C12B C13B -179.4(2) . . . . ?

C12A C13A C14A C15A -0.2(4) . . . . ?

C12A C13A C14A C17A -179.3(2) . . . . ?

C11B C12B C13B C14B 0.5(4) . . . . ?

C13A C14A C15A C16A -0.1(4) . . . . ?

C17A C14A C15A C16A 179.0(2) . . . . ?

C12B C13B C14B C15B -0.2(4) . . . . ?

C12B C13B C14B C17B 179.9(3) . . . . ?

C12A C11A C16A C15A -0.9(4) . . . . ?

N3A C11A C16A C15A 179.5(2) . . . . ?

C14A C15A C16A C11A 0.7(4) . . . . ?

C13B C14B C15B C16B 0.0(4) . . . . ?

C17B C14B C15B C16B 179.9(3) . . . . ?

C14B C15B C16B C11B 0.0(4) . . . . ?

C12B C11B C16B C15B 0.3(4) . . . . ?

N3B C11B C16B C15B 179.2(2) . . . . ?

_refine_diff_density_max   0.210

_refine_diff_density_min   -0.195

_refine_diff_density_rms   0.036

_shelx_res_file

;

TITL solve2_a.res in P-1

    solve2.res

    created by SHELXL-2017/1 at 09:16:10 on 13-Jan-2025

CELL 1.54178 8.0586 12.0229 16.9468 71.801 83.300 84.316

ZERR 4.00 0.0005 0.0008 0.0009 0.004 0.004 0.004

```
LATT 1
SFAC C H N O
UNIT 68 68 12 8
LIST 4 ! automatically inserted. Change 6 to 4 for CHECKCIF!!
TEMP 23.000
SIZE 0.110 0.180 0.200
ACTA
L.S. 14
FMAP 2
PLAN -5 0 0.00
HTAB
BOND $H
CONF
DFIX 0.89 0.01 N1A H1A N3A H3A1 N1B H1B N3B H3B1
WGHT    0.059300    0.550200
FVAR       0.31724
O1B   4   -0.034459    0.724010    0.788136    11.00000    0.07192    0.10019 =
         0.06193   -0.03338   -0.00778    0.02516
C1B   1    0.065264    0.710778    0.621297    11.00000    0.05225    0.05740 =
         0.05478   -0.02255   -0.00102   -0.00294
N1B   3    0.135104    0.650868    0.697273    11.00000    0.05883    0.06179 =
         0.05590   -0.02432   -0.00393    0.00747
AFIX  44
H1B   2    0.221384    0.598925    0.694216    11.00000   -1.20000
AFIX   0

C1A   1    0.388221    0.838801    0.307015    11.00000    0.06266    0.04924 =
         0.04546   -0.01928    0.00071   -0.00666
N1A   3    0.466603    0.780831    0.380804    11.00000    0.07183    0.05319 =
```

0.04691 -0.02484 -0.00567 0.00706

AFIX 44

H1A 2 0.526897 0.714274 0.381123 11.00000 -1.20000

AFIX 0

O1A 4 0.400216 0.907514 0.457094 11.00000 0.11593 0.05322 =

0.05281 -0.02719 -0.01686 0.01413

O2B 4 0.374476 0.490233 0.763419 11.00000 0.07522 0.07884 =

0.07581 -0.03331 -0.00379 0.01484

C2B 1 -0.041289 0.811509 0.609750 11.00000 0.06945 0.05753 =

0.06896 -0.02833 -0.00373 0.00917

AFIX 43

H2B 2 -0.072648 0.842476 0.653595 11.00000 -1.20000

AFIX 0

N2B 3 0.166992 0.599499 0.917353 11.00000 0.05266 0.06459 =

0.06103 -0.02441 -0.00273 -0.00700

C2A 1 0.283405 0.939383 0.296196 11.00000 0.08062 0.05763 =

0.05487 -0.02771 -0.00661 0.00291

AFIX 43

H2A 2 0.263555 0.974947 0.338192 11.00000 -1.20000

AFIX 0

N2A 3 0.525993 0.736892 0.597912 11.00000 0.05529 0.04987 =

0.04892 -0.02173 -0.00227 -0.00463

O2A 4 0.649447 0.586684 0.453345 11.00000 0.08642 0.06673 =

0.06434 -0.03451 -0.00372 0.01576

C3A 1 0.207847 0.987174 0.222489 11.00000 0.08842 0.05981 =

0.06669 -0.02163 -0.01682 0.00613

AFIX 43

H3A 2 0.137106 1.054880 0.215349 11.00000 -1.20000

AFIX 0

```
N3A   3    0.450971    0.829666    0.612987    11.00000    0.07519    0.04822 =
         0.04333   -0.02014   -0.00619    0.00255
AFIX  44
H3A1  2    0.408869    0.886777    0.572396    11.00000   -1.20000
AFIX   0

C4A   1    0.236217    0.935708    0.159846    11.00000    0.10018    0.07244 =
         0.05688   -0.01731   -0.01934   -0.00755
AFIX  43
H4A   2    0.185067    0.968176    0.110553    11.00000   -1.20000
AFIX   0
N3B   3    0.044992    0.668266    0.938008    11.00000    0.06116    0.07705 =
         0.05232   -0.02739   -0.00269   -0.00024
AFIX  44
H3B1  2   -0.022843    0.710824    0.899646    11.00000   -1.20000
AFIX   0

C3B   1   -0.100362    0.865381    0.532724    11.00000    0.07110    0.06490 =
         0.07428   -0.02235   -0.00780    0.01313
AFIX  43
H3B   2   -0.171518    0.932968    0.524936    11.00000   -1.20000
AFIX   0
C5A   1    0.340686    0.836034    0.170797    11.00000    0.11359    0.07669 =
         0.05043   -0.03185   -0.00802   -0.00538
AFIX  43
H5A   2    0.360502    0.801012    0.128507    11.00000   -1.20000
AFIX   0
C4B   1   -0.055535    0.820577    0.467506    11.00000    0.07590    0.08309 =
         0.06513   -0.02137   -0.00995    0.00774
```

AFIX 43

H4B 2 -0.095741 0.857515 0.415792 11.00000 -1.20000

AFIX 0

C6A 1 0.416880 0.786938 0.243762 11.00000 0.08537 0.05972 =

0.05067 -0.02768 -0.00235 0.00064

AFIX 43

H6A 2 0.487352 0.719162 0.250503 11.00000 -1.20000

AFIX 0

C5B 1 0.049092 0.720837 0.479347 11.00000 0.08326 0.08573 =

0.06224 -0.03486 -0.00689 0.01065

AFIX 43

H5B 2 0.079482 0.689996 0.435371 11.00000 -1.20000

AFIX 0

C7A 1 0.462686 0.812426 0.450866 11.00000 0.06322 0.04788 =

0.04719 -0.02234 -0.00097 -0.00312

C6B 1 0.109879 0.665634 0.555765 11.00000 0.06236 0.06386 =

0.05951 -0.02857 -0.00329 0.00924

AFIX 43

H6B 2 0.180886 0.598032 0.563068 11.00000 -1.20000

AFIX 0

C8A 1 0.537778 0.723645 0.522707 11.00000 0.05388 0.04750 =

0.04815 -0.02084 0.00027 -0.00406

C7B 1 0.088672 0.661844 0.773472 11.00000 0.05056 0.06271 =

0.05553 -0.02623 -0.00067 -0.00191

C9A 1 0.627174 0.613283 0.518375 11.00000 0.05765 0.05480 =

0.05884 -0.02502 0.00076 -0.00027

C8B 1 0.192819 0.592781 0.840748 11.00000 0.04696 0.05787 =

0.06043 -0.02524 -0.00095 -0.00606

C10A 1 0.694279 0.530931 0.596603 11.00000 0.08413 0.06172 =

0.06881 -0.02270 -0.00973 0.01557

AFIX 137

H10A 2 0.746383 0.461434 0.585391 11.00000 -1.50000

H10B 2 0.603918 0.510000 0.639554 11.00000 -1.50000

H10C 2 0.775278 0.568904 0.614690 11.00000 -1.50000

AFIX 0

C9B 1 0.335269 0.510298 0.830035 11.00000 0.05544 0.06378 =

0.06919 -0.02603 -0.00501 -0.00459

C11A 1 0.436944 0.838925 0.694435 11.00000 0.06509 0.04988 =

0.04365 -0.02001 -0.00398 -0.00844

C10B 1 0.433513 0.448872 0.902634 11.00000 0.07583 0.09562 =

0.10057 -0.03640 -0.03013 0.01855

AFIX 137

H10D 2 0.518027 0.395478 0.887158 11.00000 -1.50000

H10E 2 0.359956 0.406052 0.948394 11.00000 -1.50000

H10F 2 0.485717 0.505594 0.919022 11.00000 -1.50000

AFIX 0

C12A 1 0.539561 0.771034 0.753763 11.00000 0.06769 0.05748 =

0.05478 -0.02239 -0.00801 -0.00206

AFIX 43

H12A 2 0.621062 0.717474 0.740826 11.00000 -1.20000

AFIX 0

C11B 1 0.018963 0.676248 1.019555 11.00000 0.05898 0.07556 =

0.05203 -0.02417 0.00224 -0.01821

C13A 1 0.519376 0.783846 0.832256 11.00000 0.08548 0.06742 =

0.05117 -0.01610 -0.01526 -0.01179

AFIX 43

H13A 2 0.588667 0.737979 0.872031 11.00000 -1.20000

AFIX 0

C12B 1 0.105605 0.603785 1.083438 11.00000 0.06043 0.08019 =

0.06775 -0.02660 -0.00740 -0.01074

AFIX 43

H12B 2 0.185607 0.547444 1.073247 11.00000 -1.20000

AFIX 0

C14A 1 0.400598 0.861920 0.854138 11.00000 0.08405 0.07498 =

0.04575 -0.02544 0.00037 -0.02049

C13B 1 0.074205 0.614320 1.163217 11.00000 0.07528 0.09600 =

0.05828 -0.02373 -0.00930 -0.01962

AFIX 43

H13B 2 0.134467 0.565000 1.205763 11.00000 -1.20000

AFIX 0

C15A 1 0.299952 0.929167 0.793712 11.00000 0.08248 0.07838 =

0.06658 -0.04158 0.00280 0.00108

AFIX 43

H15A 2 0.218443 0.982636 0.806763 11.00000 -1.20000

AFIX 0

C14B 1 -0.044276 0.696237 1.180765 11.00000 0.08105 0.09967 =

0.05872 -0.03244 0.00570 -0.03007

C16A 1 0.318040 0.918531 0.713978 11.00000 0.07959 0.06549 =

0.05621 -0.02856 -0.01189 0.01008

AFIX 43

H16A 2 0.249937 0.965110 0.673885 11.00000 -1.20000

AFIX 0

C15B 1 -0.131031 0.768412 1.115590 11.00000 0.08210 0.09448 =

0.07316 -0.03633 0.00707 -0.00734

AFIX 43

H15B 2 -0.211617 0.824431 1.125768 11.00000 -1.20000

AFIX 0

```
C17A  1    0.381326    0.875810    0.940495    11.00000    0.12516    0.11525 =
         0.05483   -0.03871    0.00144   -0.02697
AFIX 137
H17A  2    0.400749    0.800695    0.981032    11.00000   -1.50000
H17B  2    0.270001    0.907308    0.951617    11.00000   -1.50000
H17C  2    0.460998    0.928243    0.943640    11.00000   -1.50000
AFIX   0
C17B  1   -0.077438    0.706771    1.266845    11.00000    0.12062    0.15090 =
         0.06425   -0.04501    0.01007   -0.03330
AFIX 137
H17D  2   -0.095076    0.630726    1.306032    11.00000   -1.50000
H17E  2   -0.175437    0.758271    1.269554    11.00000   -1.50000
H17F  2    0.016897    0.738084    1.280246    11.00000   -1.50000
AFIX   0
C16B  1   -0.100694    0.759105    1.036175    11.00000    0.07853    0.08466 =
         0.06210   -0.02862    0.00093    0.00337
AFIX  43
H16B  2   -0.160635    0.808538    0.993542    11.00000   -1.20000
AFIX   0
HKLF 4 1 1 0 0 0 1 0 0 0 1


REM  solve2_a.res in P-1
REM R1 =  0.0525 for    3664 Fo > 4sig(Fo)  and  0.0831 for all    5461 data
REM    405 parameters refined using      4 restraints

END
```

WGHT 0.0593 0.5502

REM Instructions for potential hydrogen bonds

HTAB N1B O2B

HTAB N1A O2A

HTAB C2B O1B

HTAB C2A O1A

HTAB N3A O1A

HTAB N3B O1B

EQIV $1 -x+1, -y+1, -z+1

HTAB C6B O2A_$1

HTAB C10A O2B

REM Highest difference peak 0.210, deepest hole -0.195, 1-sigma level 0.036

Q1 1 0.3695 0.4628 0.9778 11.00000 0.05 0.21

Q2 1 0.3774 0.9785 0.9289 11.00000 0.05 0.18

Q3 1 0.3225 0.4485 1.0005 11.00000 0.05 0.18

Q4 1 0.2838 0.8115 0.9800 11.00000 0.05 0.17

Q5 1 0.4435 0.3570 0.9091 11.00000 0.05 0.16

;

_shelx_res_checksum 50130

CheckCIF report

Datablock: solve2

Bond precision: C-C = 0.0035 A Wavelength=1.54178

Cell: a=8.0586(5) b=12.0229(8) c=16.9468(9)

alpha=71.801(4) beta=83.300(4) gamma=84.316(4)

Temperature: 296 K

	Calculated	Reported
Volume	1545.74(17)	1545.74(17)
Space group	P -1	P -1
Hall group	-P 1	-P 1
Moiety formula	C17 H17 N3 O2	?
Sum formula	C17 H17 N3 O2	C17 H17 N3 O2
Mr	295.34	295.33
Dx,g cm-3	1.269	1.269
Z	4	4
Mu (mm-1)	0.690	0.690
F000	624.0	624.0
F000'	625.87	
h,k,lmax	9,14,20	9,14,20
Nref	5487	5461
Tmin,Tmax	0.871,0.927	0.640,0.930
Tmin'	0.871	

Correction method= # Reported T Limits: Tmin=0.640 Tmax=0.930 AbsCorr = MULTI-SCAN

Data completeness= 0.995 Theta(max)= 66.661

R(reflections)= 0.0525(3664) wR2(reflections)= 0.1573(5461)

S = 1.021 Npar= 405

The following ALERTS were generated. Each ALERT has the format
test-name_ALERT_alert-type_alert-level.
Click on the hyperlinks for more details of the test.

Alert level C

PLAT905_ALERT_3_C Negative K value in the Analysis of Variance ... -1.974 Report
PLAT911_ALERT_3_C Missing FCF Refl Between Thmin & STh/L= 0.596 26 Report
-3 13 3, 2 14 3, 2-12 4, -9 -2 4, -8 6 5, -1 14 5,
-3-11 6, -6 10 6, 6 12 6, -7 8 7, -3 13 7, 9 0 8,
-5 11 8, -5 10 11, 6 -4 12, 6 11 12, -2 -7 13, 8 3 13,
8 4 13, -6 1 14, -6 4 14, 3 -5 15, 6 3 17, 2 11 17,
0 -2 18, 0 4 20,

Alert level G

PLAT002_ALERT_2_G Number of Distance or Angle Restraints on AtSite 8 Note
PLAT154_ALERT_1_G The s.u.'s on the Cell Angles are Equal ..(Note) 0.004 Degree
PLAT172_ALERT_4_G The CIF-Embedded .res File Contains DFIX Records 1 Report
PLAT720_ALERT_4_G Number of Unusual/Non-Standard Labels .... 2 Note
H3A1 H3B1
PLAT860_ALERT_3_G Number of Least-Squares Restraints .... 4 Note
PLAT883_ALERT_1_G Absent Datum for _atom_sites_solution_primary .. Please Do !
PLAT909_ALERT_3_G Percentage of I>2sig(I) Data at Theta(Max) Still 36% Note
PLAT969_ALERT_5_G The 'Henn et al.' R-Factor-gap value .... 2.436 Note
Predicted wR2: Based on SigI**2 6.46 or SHELX Weight 15.42
PLAT978_ALERT_2_G Number C-C Bonds with Positive Residual Density. 0 Info

---

0 **ALERT level A** = Most likely a serious problem - resolve or explain
0 **ALERT level B** = A potentially serious problem, consider carefully
2 **ALERT level C** = Check. Ensure it is not caused by an omission or oversight
9 **ALERT level G** = General information/check it is not something unexpected

2 ALERT type 1 CIF construction/syntax error, inconsistent or missing data
2 ALERT type 2 Indicator that the structure model may be wrong or deficient
4 ALERT type 3 Indicator that the structure quality may be low
2 ALERT type 4 Improvement, methodology, query or suggestion
1 ALERT type 5 Informative message, check

Validation response form

Please find below a validation response form (VRF) that can be filled in and pasted into your CIF.

```
# start Validation Reply Form
_vrf_PLAT905_solve2
;
PROBLEM: Negative K value in the Analysis of Variance ...     -1.974 Report
RESPONSE: ...
;
_vrf_PLAT911_solve2
;
PROBLEM: Missing FCF Refl Between Thmin & STh/L=    0.596         26 Report
RESPONSE: ...
;
# end Validation Reply Form
```

It is advisable to attempt to resolve as many as possible of the alerts in all categories. Often the minor alerts point to easily fixed oversights, errors and omissions in your CIF or refinement strategy, so attention to these fine details can be worthwhile. In order to resolve some of the more serious problems it may be necessary to carry out additional measurements or structure refinements. However, the purpose of your study may justify the reported deviations and the more serious of these should normally be commented upon in the discussion or experimental section of a paper or in the "special_details" fields of the CIF. checkCIF was carefully designed to identify outliers and unusual parameters, but every test has its limitations and alerts that are not important in a particular case may appear. Conversely, the absence of alerts does not guarantee there are no aspects of the results needing attention. It is up to the individual to critically assess their own results and, if necessary, seek expert advice.

Publication of your CIF in IUCr journals

A basic structural check has been run on your CIF. These basic checks will be run on all CIFs submitted for publication in

IUCr journals (*Acta Crystallographica*, *Journal of Applied Crystallography*, *Journal of Synchrotron Radiation*); however, if you intend to submit to *Acta Crystallographica Section C* or *E* or *IUCrData*, you should make sure that full publication checks are run on the final version of your CIF prior to submission.

Publication of your CIF in other journals

Please refer to the *Notes for Authors* of the relevant journal for any special instructions relating to CIF submission.

PLATON version of 19/12/2024; check.def file version of 19/12/2024

Datablock solve2 - ellipsoid plot

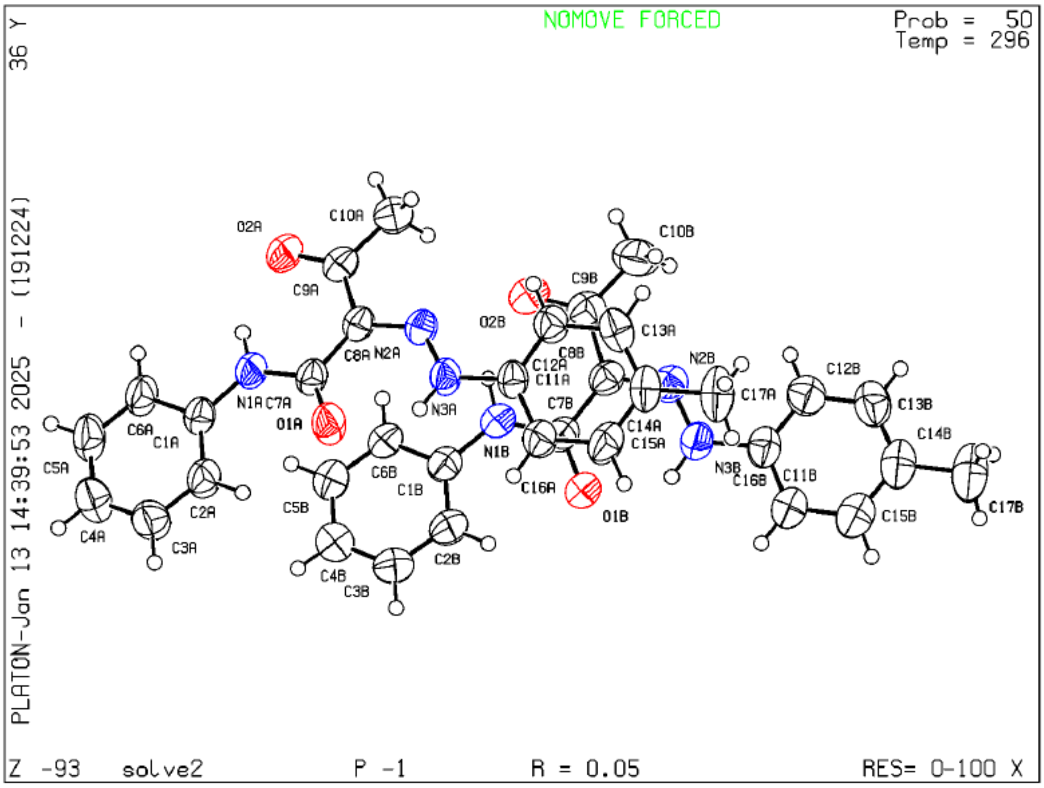

Supplement: Supplementary file 1 — Supplementary Material [file OPEN-14-e202500276-s001.pdf]
